# Addressing social determinants of health and equity in early childhood: a qualitative document analysis of national policies in Ecuador

**DOI:** 10.1186/s12939-026-02891-2

**Published:** 2026-05-29

**Authors:** Grace Navarrete Chávez, Alex García Gutiérrez, Diego Andrade Ortíz, Daniel Kopasker, Ruth Dundas, Srinivasa Vittal Katikireddi

**Affiliations:** 1 School of Health and Wellbeing, University of Glasgow, University Avenue, Glasgow, G12 8QQ UK; 2 Universidad Internacional del Ecuador (UIDE), Av. Simón Bolívar y Av. Jorge Fernández, Quito, 170505 Ecuador

**Keywords:** Early childhood, Health policy, Social determinants of health, Health equity, Document analysis

## Abstract

**Background:**

Early childhood health is a major policy priority in Ecuador, yet systematic analyses of relevant policies are limited. This study analyses how Ecuadorian national-level policies (2000–2024) have addressed social determinants of health (SDH) in early childhood and promoted health equity. We map the development and scope of the past and present national policy documents across sectors to identify gaps, strengths and priorities within the policy landscape.

**Methods:**

Following a systematic search, we conducted a qualitative analysis of 29 policy documents using the READ approach (ready the materials, extract data, analyse, and distil findings), with thematic analysis. We combined deductive codes related to governance instruments and six SDH living-conditions domains (healthcare, health services, early education, social protection, childcare, and housing) with inductive codes that emerged during analysis. NVivo software supported data management and theme development, paying attention to contradictory evidence. An advisory board reviewed the interpretation of findings.

**Results:**

Early policy documents focused on sector-specific interventions, whereas later policies reflected more holistic approaches. Recent policies strongly promoted intersectoral collaboration but rarely specified clear financing or evaluation mechanisms. All six SDH domains analysed were represented, but attention was unbalanced: healthcare and health services dominated, while housing, early education and childcare were under-represented. Applying a policy typology, most policies were universal or targeted; only one was redistributive, and the use of proportionate universalism was uneven. Commitments to equity became more prominent over time through recognising vulnerable groups and intercultural approaches, yet concrete recommendations on intersectionality, territorial equity and community-based planning remained scarce.

**Conclusion:**

Embedding social determinants of health in policy design, underpinned by robust governance, effective intersectoral collaboration, sustainable financing, and locally responsive strategies, is essential, particularly for children facing intersecting disadvantages and those living in underserved territories. Stronger evidence on governance structures, implementation processes, and equity-oriented evaluations using lower-level territorial health indicators is crucial not only for Ecuador but also for other low-and middle-income countries seeking to advance equity in the earliest years and across future generations.

**Supplementary Information:**

The online version contains supplementary material available at 10.1186/s12939-026-02891-2.

## Background

Inequities in health in the early years of life often persist into adulthood, perpetuating cycles of poverty and social exclusion [1, 2]. Improving early childhood health is pivotal not only for enhancing human capital but also for increasing long-term societal benefits [3–5].

The early period of life, particularly the first 1000 days, has been identified as the most valuable opportunity for implementing policies to improve child and population health [6]. Emerging evidence also highlights the importance of the subsequent 1000 days (ages 2–5) as another crucial period for shaping healthy life trajectories [7]. Together, these stages, the prenatal period through the age of five, represent the window through which policies can most effectively determine pathways for child health equity.

Global early childhood frameworks recognise the importance of early-life social determinants of health and support calls to place children at the centre of the Sustainable Development Goals (SDGs) agenda [8]. For instance, the Nurturing Care Framework promotes coordinated policy action across five domains (health, nutrition, responsive caregiving, safety and security, and early learning) to promote healthy development in young children and advance relevant child-related SDG goals [9, 10].

To capture how policies address these critical stages, we used the social determinants of health (SDH) framework, which emphasises the importance of social and environmental factors that influence well-being and health outcomes [11, 12]. In regard to children, the SDH framework helps understand how the interplay between parents, communities and macrolevel structures shapes child health [13]. Social determinants of child health range from fixed individual characteristics to modifiable aspects and living conditions that influence children’s health directly and indirectly via their parents and carers. It also includes broader factors such as political, cultural, commercial and economic conditions [14].

While social determinants of child health operate across all contexts, within the SDGs agenda, low- and middle-income countries (LMICs) represent a critical focus area with nearly 250 million children under five at risk of not achieving their full developmental potential [9, 15]. Although significant progress has been made in the establishment of early childhood policy frameworks in LMICs, policy effectiveness remains inconsistent due to factors such as limited funding, inadequate monitoring and evaluation mechanisms, weak intersectoral collaboration, and a lack of equity-based policy recommendations [16, 17].

These challenges are particularly relevant to Latin America, where significant health inequities persist between and within countries [18, 19]. Ecuador reflects this broader regional context. A major socioeconomic crisis in the late 1990s led to widespread impoverishment with implications for children’s health and living conditions [20–23]. In response, an emergency social plan emerged in 2000, within a context of political, economic, and institutional instability [24].

A second turning point followed the introduction of the 2008 Constitution, which recognised health as a human right and supported new approaches to equity and social inclusion [25, 26]. In 2012, the Comprehensive Family, Community, and Intercultural Healthcare Model (MAIS-FCI, by its Spanish acronym) reorganised service organisation, management, financing, and provision around primary health care [23, 26]. In parallel, broader institutional reforms, particularly in social protection, strengthened the state’s role in planning child-focused policies [24]. From 2018 onwards, fiscal austerity constrained the continuity of social policies, and from 2020, the COVID-19 pandemic further shaped the context in which early-childhood policies have been formulated and implemented [23].

In this context, from 2000 until 2024, a wide range of early childhood policy initiatives were developed, focusing on different social groups and addressing various topic areas. Although considerable progress has been made in reducing national child mortality rates over time, this health equity aggregated indicator alone does not necessarily capture persistent health inequities affecting disadvantaged children in Ecuador across lower territorial levels [27–29].

Since policy design in Ecuador occurs primarily at the national level, defining the frameworks and resources that shape early childhood health, we map the development and scope of the past and present national policy documents across various sectors to identify gaps, strengths and priorities. While a variety of sources have been suggested for policy analysis, this paper focuses on national government policy documents, as they are key elements of policy processes and capture articulated policy intentions [30].

To the best of our knowledge, prior policy analyses of early childhood health in Ecuador and Latin America have tended to focus on specific sectors (e.g., nutrition, social security, health services) or on selected targeted policies, with limited assessment of equity across the broader policy landscape. This study addresses this gap by providing a longitudinal mapping of early childhood-related policies and systematically analysing the extent to which policies address the SDH and articulate equity-oriented design and governance.

The analysis was guided by three research questions: (1) What policies aimed at improving early childhood health outcomes were documented in Ecuador between 2000 and 2024? (2) How did policies evolve during this period? (3) What recommendations are stated in public policy documents to address the social determinants of health and promote health equity in early childhood?

## Methods

Our study used qualitative document analysis to identify, describe, and analyse official policy documents related to early childhood health in Ecuador. We followed the four‑step READ approach for this purpose [31]. This method comprises (1) ready the materials (defining criteria and collecting documents), (2) extract data (systematically identifying relevant content), (3) analyse data (coding and theme development), and (4) distil findings (synthesising results and interpreting meaning). This structured method facilitates the systematic search of policy documents and supports transparency in qualitative analysis.

A thematic analysis was conducted using a hybrid strategy, incorporating both inductive and deductive coding. Coding and theme organisation were managed in NVivo (version 14) [32] and an advisory board provided feedback on the interpretation of the findings.

As a theoretical framework, we focused on the SDH intermediate living-conditions layer because policy documents most often specify actionable measures at this level. This allowed us to assess implementability by examining whether policies identify concrete levers, responsible actors, and governance mechanisms, while recognising that upstream structural determinants are typically treated as contextual background in policy texts.

### Step 1: Ready the materials

#### Eligibility criteria

We applied eligibility criteria across four dimensions: policy level, target population, document type, and content validity (see Table 1). We included documents explicitly relevant to early childhood health (antenatal to age five). Content validity was assessed against the study aims, retaining documents that contained policy document recommendations relevant to the six SDH living-conditions domains and equity. We excluded purely administrative documents (e.g., internal procedures), clinical guidelines, subnational-only instruments, duplicates and superseded versions where a newer official version was available.

**Table 1:** Eligibility criteria for selecting national early childhood policy documents in Ecuador (2000–2024)

Criteria	Inclusion	Exclusion
Policy level	National level	Range at provincial, canton or parish level or related only to a specific institution
Target Population	Policy documents addressing early childhood (0–5 years, including antenatal period) or broader documents that also cover this age group	Policy documents not covering early childhood (0–5 years, including antenatal period)
Type of document	• Laws and national policies • Services and strategies • Plans, programmes, and projects	• Administrative procedures • Broader contextual laws • International agreements • Clinical guidelines
Content validity	• Related to six SDH categories • Non-emergency situations or special protection of rights • Recommendations aimed at improving early childhood health • Latest published version	• Not related to six SDH categories • Emergency or temporary situations, or for special protection of rights* • No recommendations for early childhood health • Previous versions of the policy document already included.

Note: SDH = Social Determinants of Health. Broader policy documents were included only when they also covered early childhood (0–5 years, including antenatal). *Special protection policies, such as alternative care (foster or institutional care), were excluded as they address specific responses rather than broad early childhood health determinants. Inclusion of the latest document version refers to the most recent published version available at the time of review

#### Policy document sources and search terms

To retrieve policy documents, the lead author (GNCH) searched Lexis (an Ecuadorian subscription-based legal repository) [33]. Although legislation and normative instruments are publicly available via Ecuador’s Official Registry website (Registro Oficial), its online interface offers limited advanced search and filtering. In contrast, Lexis enables indexed full-text searching and filtering across the study period.

While Lexis compiles electronic copies of national legislation and normative instruments (including ministerial regulations) from the Official Registry, we also searched ministry websites to ensure no relevant policy documents were missed, particularly non-legislative materials (e.g., strategies, plans, and institutional guidance). Given variability across ministry websites and limitations in search functionality, these searches combined available site-search tools with manual navigation. In addition, a targeted Google Advanced and grey literature search was planned a priori and conducted subsequently to maximise retrieval sensitivity, following sequential searches of Lexis and institutional websites. Given that all the policy documents were issued in Ecuador, we restricted the search language to Spanish. The search strategy was conducted from October 2024 to December 2024 (Supplementary Method 1). Search terms used included: open terms using variations of “childhood”, “children”, and “infancy” in Spanish; and category search using terms related to six living condition determinants, adapted from the Social Determinants of Child Health Framework [14], namely early education, healthcare, health services, social protection, childcare, and housing.

#### Search strategy rationale

The search aimed to collect national-level policy documents published from 2000 to 2024 that might affect early childhood health, defined here from the antenatal period through the age of five. The starting point of 2000 was selected because it marks the onset of sustained national policy responses affecting early childhood following the late-1990s socioeconomic crisis, allowing us to capture subsequent policy developments through to 2024.

### Step 2: Data extraction

Two reviewers (GNCH and AGG) screened records across three sequential phases: (i) title screening, (ii) text skimming, and (iii) full-text eligibility assessment. Identified and labelled records from each search source were exported to separate Excel files and consolidated into two datasets: one combining Lexis and ministry website records, and a second for Google advanced search/grey literature records. Duplicates were removed from the consolidated datasets, and the remaining documents screened. Screening decisions were recorded using structured Excel templates (multiple dated worksheets per phase), with exclusions labelled and tracked; Excel filtering and sorting functions facilitated title screening and management across phases. Full-text documents were downloaded from sources as PDF files and shared via a secure cloud folder for the textual data review.

GNCH and AGG worked in parallel (asynchronously), and both reviewers assessed all records at each phase using identical copies of the phase-specific dataset and policy documents. After each phase, GNCH compiled both reviewers’ files into a single comparison spreadsheet to identify agreements and discrepancies. Discrepancies were resolved through discussion in a videoconference meeting, and an updated consensus dataset was produced and redistributed to both reviewers for the subsequent phase. No substantial disagreements arose that required adjudication by a third reviewer. Consensus meetings were held weekly (four meetings in total, February-early March 2025), three meetings followed each screening phase for the Lexis/institutional websites dataset, and one meeting reconciled the Google/grey literature dataset.

### Step 3: Qualitative document analysis

#### Coding process

Following familiarisation with all selected documents, GNCH conducted line-by-line manual coding, applying the deductive codes and generating additional inductive codes. Codes were iteratively reviewed, redundant codes removed, and grouping conceptually related codes, resulting in a consolidated set of 67 codes organised into 14 higher-order themes. Full code definitions and illustrative excerpts are provided in Supplementary Tables 1, and the coding process is summarised in Supplementary Fig. 1.

Initial coding was done manually to help familiarise the lead researcher with the data and to develop the coding framework. After the coding framework was finalised, NVivo version 14 was used to code all available data to support systematic organisation and thematic grouping. When developing findings, we paid attention to contradictory data and the context of the policy document from which data arose, retaining these cases to refine themes where appropriate.

To summarise the policies (RQ1), we developed a table describing the characteristics of the policy documents and components stated (manifest content analysis). Thematic analysis was used to characterise the evolution of policies (RQ2) and to create a timeline of the policies. For RQ3, recommendations were mapped to the six social determinant categories and three inductively identified equity-related themes.

In relation to equity, we coded actions recommended in policies according to Benach’s typology of policy approaches to reducing health inequalities [34], which distinguishes between gap-focused universalism, pure targeting, redistribution, and proportionate universalism. This framework was applied after the initial coding process and helped situate the findings within international debates on how equity is operationalised in policy design. We additionally coded whether policies targeted specific vulnerable groups and whether they adopted an intercultural approach.

#### Translation and verification

Policy document excerpts from the codebook used for analysis and reporting were translated from Spanish into English by the lead author (GNCH). To ensure accuracy and conceptual equivalence, translations were reviewed during two dedicated verification meetings held in May 2025 with a bilingual researcher (DAO). These meetings focused on assessing the precision of translated excerpts, their contextual meaning, and consistency with the original policy language. Translation decisions and revisions were documented using an Excel comparison template that recorded original Spanish text, translated excerpts, and agreed adjustments.

#### Reflexivity and analytical rigour

The lead author, GNCH, maintained a detailed fieldwork diary throughout the analysis process to document iterative analytic decisions (e.g., eligibility judgements, code refinements, and emergent interpretations). As a researcher with a professional background in health policy practice in Ecuador, GNCH provided contextual insight that shaped the reading and understanding of policy documents, particularly regarding the health system, service delivery, and institutional frameworks, including terminology and sectoral actors described in the texts. To strengthen reflexivity, the research team (GNCH, SVK, DK, RD) held four monthly meetings (March-June 2025) to review coding decisions and theme development, discuss emerging interpretations, and refine the narrative synthesis of results.

#### Coding framework

The coding framework was developed by the lead author (GNCH), drawing on the literature on key components of policy documents. Four deductive descriptive codes were defined to capture cross-cutting governance features: family and community participation, intersectoral collaboration, financing, and monitoring and evaluation. Code definitions were specified a priori; for example, intersectoral collaboration was defined as references to formal coordination mechanisms, shared responsibilities, or joint actions across sectors (e.g., health, education, social protection).

In parallel, we applied six deductive codes from the SDH’s living conditions layer, defined as the modifiable settings in which children are born and grow that are most amenable to policy action, namely: early education, healthcare, health services, social protection, childcare, and housing [14].

During the analysis, additional inductive codes were generated to capture emergent issues that were not fully anticipated in the initial framework. These inductive codes captured, for example, references to an intercultural approach and intersectional dimensions of vulnerability.

### Step 4: Distil findings

#### Advisory board consultation

An advisory board of early childhood health policy experts was convened to strengthen interpretive credibility. Members were purposively selected using predefined criteria to ensure coverage across the six SDH living-conditions domains and complementary policy expertise (e.g., national-level experience, at least 10 years of professional trajectory, and evidence of relevant outputs such as scientific publications or contributions to national technical reports). Ten experts were invited (balanced by gender); seven accepted and were scheduled, and five attended. Ten days before the meeting, preliminary results (in Spanish), including summary tables and figures, were circulated. A two-hour online consultation (15 July 2025) discussed the review process and emerging interpretations and checked for any missing electronically available national policy documents; no additional eligible documents were proposed. Feedback was documented in anonymised notes and used to refine interpretation; no further rounds were required.

#### Synthesis approach

We synthesised findings through a thematic synthesis: coded content was summarised into themes aligned with the research questions and presented descriptively with illustrative policy excerpts. This approach was selected because selected policy documents varied substantially in scope, format, and level of detail, making thematic summarisation with narrative integration the most appropriate synthesis strategy.

## Results

**Fig. 1:**
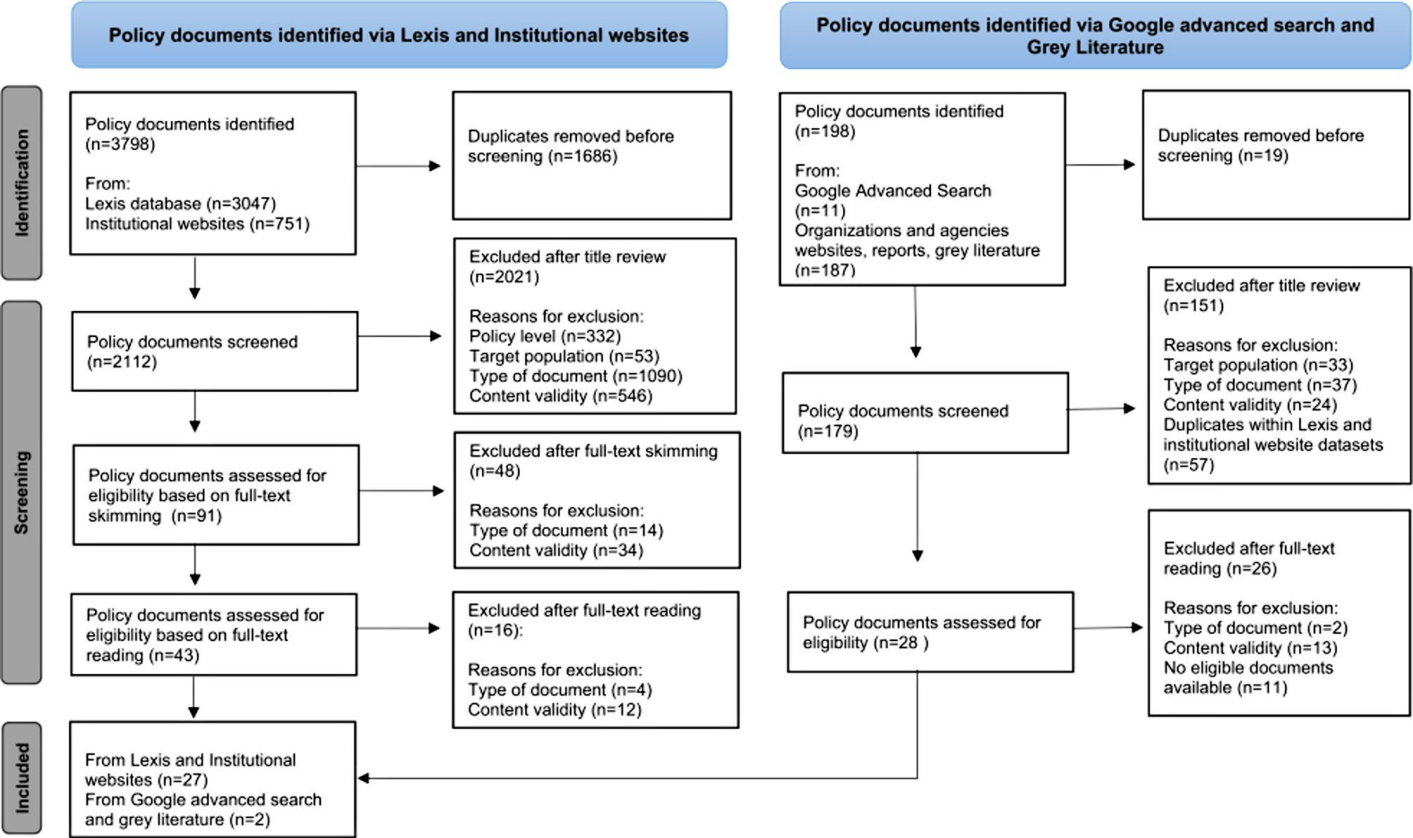
Flow diagram detailing the selection process of the policy documents included in the study. Note: n = number of policy documents. An additional skimming stage was included before full-text assessment. 11 full-text documents were unavailable in electronic format

Across Lexis and official ministerial websites, 2,112 potentially relevant documents were identified. Following title screening and rapid text screening (skimming), a subset of 43 documents underwent full-text eligibility assessment, and 27 were finally selected. In parallel, two additional policy documents that met the eligibility criteria were identified through targeted Google and grey literature searches. This process resulted in a final set of 29 policy documents included in the analysis (see Fig. 1).

### Description of policy documents

Among the 29 national-level policy documents included, most were led by the Ministry of Public Health (*n* = 14) or the Ministry of Economic and Social Inclusion (*n* = 8). Three policies were led by the Ministry of Labour; although framed under parental work, they contained provisions for childcare and healthcare and were therefore included in the review. The remaining documents were led by the Ministry of Housing (*n* = 3) and the Technical Secretariat (*n* = 1).

Although most of these documents were published or updated between 2018 and 2024, they represent the most recent versions of long-standing policies, some with origins dating back to the early 2000s. This strategy of analysing the latest (at the time of writing) version of documents ensured a historical overview while capturing current policy frameworks.

The selected documents encompass a range of legal and strategic instruments, including laws, operational manuals, technical standards, strategic plans, and programmes. Their objectives and target population vary widely from maternal and child healthcare initiatives to early education, social protection, and child service delivery. Table 2 shows that the analysed policy documents span multiple sectors and target populations and that the four cross-cutting governance features are unevenly specified: family/community participation (*n* = 10), intersectoral collaboration (*n* = 13), financing (*n* = 8), and monitoring and evaluation (*n* = 17).

**Table 2:** Description of national early childhood policy documents analysed (2000–2024)

Lead Institution	Year	Title of the policy document	Objective (summary)	Target Population	Family and Community Participation	Intersectoral collaboration	Financing	Monitoring and evaluation
Ministry of Public Health	2006	Law on Free Maternity and Child Health Care	Financing and coverage for free maternal and child health care	Pregnant women Children < 5 yrs	■	■	■	
	2018	Procedures for the Healthcare of Prevalent Childhood Illnesses	Reduce infant mortality by managing the prevalent health problems	Children < 5 yrs				
	2011	Zero Malnutrition Project	Eliminate child malnutrition in selected parishes	Children < 1 yr			■	
	2018	Comprehensive Child Healthcare Manual	Reduction of morbidity and mortality in children	Children < 5 yrs*				
	2013	Standard for Essential Obstetric and Neonatal Healthcare	Standardise practices to improve maternal and neonatal healthcare	Pregnant women neonates	■	■		
	2018	Intersectoral Plan for Food and Nutrition	Achieve adequate nutrition and human development	Pregnant women Children < 5 yrs*		■	■	■
	2023	Manual for Monitoring Nutritional Status of Iodine	Control of the nutritional status of iodine to prevent iodine disorders	Pregnant women				■
	2019	Manual of Vaccines for Immunopreventable Diseases	Provide guidelines for vaccine use and administration in the population	Children < 5 yrs*				■
	2023	Manual on the Articulation of Practices and Knowledge of Ancestral Midwives	Inclusion of traditional ancestral midwives in National Health System	Pregnant women neonates	■			■
	2014	Neonatal Metabolic Screening Test in Health Units	Implementation of the mandatory metabolic screening test	Neonates				
	2019	Strategy for the Elimination of Mother-to-Child Transmission of HIV, Syphilis, Hepatitis B, And Chagas	Eliminate mother-to-child transmission of HIV and other relevant diseases.	Pregnant women Neonates				■
	2024	Healthcare for Pregnant Women and Neonates in Mobility Conditions	Comprehensive healthcare in situation of human mobility and migration	Pregnant women Neonates				
	2009	National Breastfeeding Policy	Guarantee and promote breastfeeding rights for children	Children < 3 yrs	■	■		
	2008	National Plan for Reducing Maternal and Neonatal Mortality	Improve access, opportunity, continuity and quality of healthcare	Pregnant women Neonates				
Ministry of Economic and Social Inclusion	2023	Family Care Service Growing with Our Children	Promote the comprehensive development of children	Pregnant women Children < 3 yrs	■	■		■
	2023	Technical Standard for the Child Development Centre Service	Provide integrated care through early learning, health, nutrition, and protective environments	Children 1–3 yrs	■	■		■
	2019	National Plan for the Prevention of Violence Against Children and Adolescents	Eliminate the causes of violence affecting children and adolescents	Children < 5 yrs*		■		■
	2018	Human Development Benefit aimed at Beneficiaries with Minor Children	Regulate the variable component in the Human Development Benefit	Children < 5 yrs*				■
	2022	Benefit for Children in Case of Violent Death of their Mother	Benefit for children in orphanhood due to the mother’s violent death	Children < 5 yrs*				
	2023	Family Support Modality for Beneficiaries of the Human Development Benefit with Variable Component	Family support service for users of the Human Development Benefit	Children < 5 yrs*	■	■		■
	2010	Joaquín Gallegos Lara Benefit for People with Disabilities	Benefit for people with severe disabilities and catastrophic diseases	Children < 5 yrs*		■	■	■
	2007	“Aliméntate Ecuador” Programme	Provide nutrition and healthcare for children in poverty	Children 3–5 yrs				
Ministry of Labour	2024	Breastfeeding Support Rooms in the Workplace	Implement breastfeeding support rooms in workplaces	Children < 2 yrs			■	■
	2023	Childcare for Children of Public Servants	Regulate the granting of the benefit of childcare	Children < 5 yrs			■	■
	2023	Organic Law on the Right to Human Care	Protect and regulate the right of working people to childcare	Pregnant women Children < 5 yrs	■	■		■
Ministry of Housing	2024	Regulation for Access to Housing Subsidies and Incentives	Access to social housing subsidies and incentives	Pregnant women Children < 5 yrs*				
Technical Secretariat	2020	Comprehensive Child Development Operational Manual “Misión Ternura”	Promote child development and well-being through intersectoral services	Pregnant women Children < 5 yrs	■	■		■
	2020	National Strategy Ecuador Grows without Malnutrition	Prevention and elimination of chronic child malnutrition	Pregnant women Children < 2 yrs	■	■	■	■
	2022	1000 Days Benefit	Guarantee a minimum level of consumption and promote the use of child services	Pregnant women Children < 2 yrs		■	■	■

Note: Overview of 29 national early childhood policy documents, grouped by lead institution and presented in the order identified through the systematic search. (∗) Policy documents also covered other target age groups beyond early childhood. The table indicates with a black square (■) whether each policy incorporated recommendations on key components such as financing, monitoring, family and community participation, and intersectoral collaboration

Our findings revealed that certain components of policy design were frequently referenced across documents, including family and community participation, intersectoral collaboration, financing mechanisms, and monitoring and evaluation. These components are described below via illustrative examples from the policy texts.

The policy documents analysed highlight the importance of community and family participation. Three reasons were identified for ensuring participation: first, monitoring and surveillance of the correct implementation and execution of the policy; second, being active actors in the protection of rights; and third, promoting co-responsibility in children’s development and well-being. Below is an example:

*The CDIs* (Spanish abbreviation for Child Development Centres) *must form a family committee*,* which will focus on the comprehensive protection of children through the effective exercise of their rights…*

(Technical standards of the Child Development Centre service, 2023)

Intersectoral collaboration was also often highlighted; the social protection sector is particularly eager to adopt this approach. In the most recent period (2018–2024), child health policies, especially when focused on child development and child nutrition, operationalise intersectoral mechanisms to enhance impact through the involvement of multiple sectors.

*In the Cantonal Intersectoral Technical roundtables*,* the commitment of the various institutional actors is promoted*,* strengthening the shared leadership of the local governments*

(Comprehensive Child Development Operational Manual “Misión Ternura”, 2020)

Financing statements were rarely mentioned in policy documents. Of the 29 documents analysed, eight policies specified the sources of funding, sustainability, or mechanisms for allocating budgets, and these provided only minor details.

*The monthly inclusions and the total population covered by this cash transfer will be based on the institutional budget allocation made by the governing body of public finances for a fiscal year*,* subject to available resources*

(1000 days Benefit, 2022)

While policy documents include general statements about monitoring and evaluation mechanisms and increasingly mention information systems and individual monitoring, they typically omit specific goals, indicators, timelines or methodologies.

*Follow-up and monitoring. The national health*,* social welfare*,* education*,* and labour authorities will follow up and monitor the implementation of breastfeeding support rooms and child development care centres*,* according to the scope of their competences*

(Breastfeeding support rooms in workplaces, 2024)

Taken together, the policy documents outline foundational components of policy design, which vary across policy types and periods. Building on this descriptive map, the following section examines how priorities, instruments, and policy intentions have shifted over time.

### Evolution of policies

**Fig. 2:**
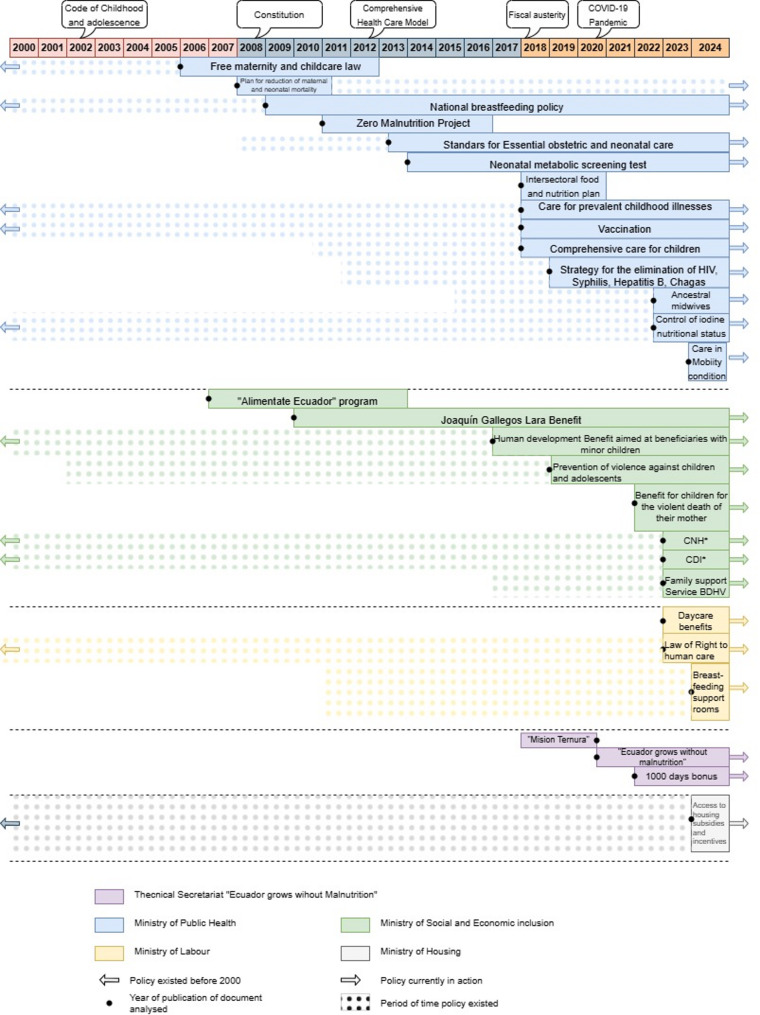
Timeline of national early childhood policies in Ecuador (2000–2024) included in the analysis. Note: Policies are shown by year of publication or latest update, illustrating their temporal distribution and institutional leadership. Periods are differentiated by color: 2000–2007 (political instability), 2008–2017 (expansion of policies), 2018–2024 (recent updates)

As illustrated in the policy timeline (Fig. 2), we identified three distinct periods of policy evolution. The first period (2000–2007) was characterised by political and institutional instability in the aftermath of Ecuador’s profound socioeconomic crisis. Second, 2008–2017, was distinguished by a restructuring of the state and public institutions, greater investment in social programmes, increasing health coverage, and the introduction of the new Constitution and Integrated Health Care Model (MAIS-FCI). Third, from 2018 to the present has been defined by the increasing fiscal constraints and the impacts of the COVID-19 pandemic.

Across the three periods analysed, Ecuadorian policies have evolved from basic interventions and isolated health-sector actions (health-centred approach) to more comprehensive approaches addressing various social determinants of child health. Nutrition policies have been more consistent in adopting recommendations aligned with this logic, for example:

*Strengthen and generate interventions that have an impact on the determinants of health*,* focused on health promotion*,* social protection*,* food security and sovereignty and water-sanitation*

(Intersectoral Food and Nutrition Plan, 2018)

The rights-based approach has evolved since children were positioned as a priority group in the Childhood and Adolescence Code (2002). This broad policy document serves as a framework for all policies to guarantee the right to the highest attainable standard of physical, mental, psychological, and sexual health. Since then, all Ecuadorian children have been considered subjects of rights, and the rights-based approach has been increasingly emphasised across all policies as a transversal axis. In addition, the 2008 Constitution introduced new approaches, such as intercultural, gender, and intergenerational, which have been embedded into policy documents reflecting a more inclusive understanding of equity and rights in policy.

*For the Family Accompaniment Service, the intergenerational approach implies promoting the participation of all its members in families, without age being an exclusion factor; ensuring that each person exercises their rights considering the stage in the life cycle in which they find themselves*

(Technical Standard for Family Support Modality for Beneficiaries of the Human Development Benefit with Variable Component, 2023)

Regarding the evolution of financing, Ecuador has historically been catalogued as an LMIC with limited public funding sources. There is a long history of centralised management through the Ministry of Finance. However, allocations of budgets for early childhood during 2000–2007 were carried out under different mechanisms, such as demand-based financing mechanisms, budgetary transfers to third parties, pre-allocations, and competitive funding schemes. Furthermore, several health and social interventions received financial support from international organisations and agencies. To illustrate local financial management in this period, the main law on maternal and child health assigned responsibility to local governments with permanent and specific funding sources.

*Local Solidarity Health Funds shall be created in each municipality, which will receive financial resources from the Solidarity Health Fund, to ensure the application of the law*

(Law on free maternity and child health care, 2006)

During the second period of analysis (2008–2017), financing was strongly centralised, and new social programmes were implemented with high public spending and mainly used the general state budget.

*The benefit will be delivered through the budget administered by the Vice Presidency of the Republic in coordination with the Ministry of Finance*,* which will implement the necessary IT tool*

(Joaquín Gallegos Lara Benefit in favour of people with disabilities, 2010)

A period of fiscal austerity became evident in 2018, and many policies remained in place but operated with varying levels of coverage. Although the country faced serious financial challenges, aggravated by the economic impact of the COVID-19 pandemic, the government reaffirmed its commitment to reducing chronic child malnutrition, a prioritised public health problem in the policy agenda, by financing an intersectoral national strategy.

*The governing body of Public Finances will be responsible for the budgetary allocation of the “prioritised package” established in Article 2 of this Decree* (Article 2 refers to specifications of the prioritised package, including a set of goods and services intended to care for pregnant women and children under 24 months of age). *The current budget allocation for these services may not be subject to reduction*

(National Strategy Ecuador Grows without Malnutrition, 2020)

Concerning monitoring and evaluation, policy documents from the first period analysed rarely included structured evaluation mechanisms or clear procedures for data collection and reporting. Limited attention was given to how policy impacts would be assessed. Nonetheless, in the health sector specifically, basic monitoring activities were more established during this period, primarily through manual routine data collection and standardised, worldwide-recognised health indicators and periodic measurements.

*The measurement will be carried out according to the indicator in the periods: monthly*,* quarterly*,* semi-annually*,* annually and at the end*

(Plan for the Reduction of Maternal and Neonatal Mortality, 2008)

Later, the evolution of monitoring and evaluation was characterised by the introduction of electronic information systems, which enable more efficient data collection. In the most recent period (2018–2024), policy documents highlighted incorporating individual-level data to track children’s social and health trajectories, as well as data linkage strategies. For example, cross-sectoral data integration is mentioned in the National Strategy against Chronic Child Malnutrition, allowing longitudinal individual monitoring through linked administrative records from multiple institutions. In the social protection sector in particular, individual monitoring has become central to service follow-up for beneficiaries, as specified below:

*The territorial technical coordinator of the MIES or whoever takes the place of the public-private institution, with the support of the family educator, must carry out the individual monitoring of the life trajectory of each child and pregnant woman to all the care provided that allows them to achieve their entitlements effectively and at the right time*

(Family Care Service Growing with our Children, 2023)

With respect to the evolution of equity and social inclusion, universal policies have consistently aimed to ensure access to essential services for all children, regardless of background. However, during the first period analysed, universal access to child services was limited. The following excerpt shows that not all children had access to initial education during this period, and that some essential service policies were targeted primarily at specific age groups or children living in poverty.

*The Aliméntate Ecuador Programme, as an entity affiliated with the Ministry of Social Welfare, will carry out its operations by serving poor children aged three to five years and eleven months who do not attend educational institutions*

(Aliméntate Ecuador Programme, 2007)

As mentioned before, adopting the new Constitution in 2008 marked a turning point for free health coverage for Ecuadorians, with children as a priority group. This macro legal framework also expanded the scope to include vulnerable groups not previously considered in policy, such as children with severe disabilities, those with catastrophic and rare diseases, and those living with HIV/AIDS. In more recent years, this scope has extended according to the new realities of the country, incorporating groups such as those experiencing orphanhood because of violence and migrant children.

*Every newborn child of a mother who is a refugee*,* asylum seeker*,* returnee*,* migrant*,* internally displaced person*,* and/or stateless person must receive quality and kind care in all health facilities of the Integrated Public Health Network. Health care is completely free*

(Care of pregnant women and newborns in mobility conditions, 2024)

An intercultural approach began to be progressively integrated into policy statements, particularly from the second period of analysis (2008–2017) onwards. This approach refers to the recognition and respectful inclusion of cultural diversity within the design and delivery of public services. In the health sector, this has translated into institutional recognition of ancestral healing practices alongside conventional biomedical care, most notably through the integrated healthcare model MAIS-FCI itself and the publication of specific policy instruments such as the Manual on the Articulation of Practices and Knowledge of Ancestral Midwives (2023).

*Institutional childbirth care by ancestral midwives will be subject to beneficial practices of ancestral midwifery: use of medicinal plants*,* ancestral techniques*,* free-position childbirth*,* immediate attachment*,* timely clamping of the umbilical cord*,* breastfeeding in the first hour of life*,* among others*

### Policy document recommendations for addressing the Social Determinants of Health

Across the policy documents reviewed, multiple recommendations varied considerably in scope and emphasis, from policies covering specific determinants to integrated, multisectoral interventions that address multiple determinants simultaneously. Table 3 maps the six SDH living-conditions domains addressed in policy document recommendations, highlighting areas of concentrated attention (healthcare in 23 documents) and domains that are comparatively under-addressed (early education and housing in 4 documents, respectively).

**Table 3:** Social determinants of health (SDH) domains addressed within Ecuadorian early childhood policies

Lead Institution	Policy Document	Early Education	Health care	Health Services	Social Protection	Child care	Housing
Ministry of Public Health	Law on Free Maternity and Child Health Care		■	■			
	Procedures for the Healthcare of Prevalent Childhood Illnesses		■	■			
	Zero Malnutrition Project		■		■		
	Comprehensive Child Healthcare Manual		■	■			
	Standard for Essential Obstetric and Neonatal Healthcare		■	■			
	Intersectoral Plan for Food and Nutrition		■	■	■		■
	Manual for Monitoring Nutritional Status of Iodine		■				
	Manual of Vaccines for Immunopreventable Diseases		■				
	Manual on the Articulation of Practices and Knowledge of Ancestral Midwives		■	■			
	Neonatal Metabolic Screening Test in Health Units		■	■			
	Strategy for the Elimination of Mother-to-Child Transmission of HIV, Syphilis, Hepatitis B, And Chagas		■	■			
	Healthcare for Pregnant Women and Neonates in Mobility Conditions		■	■			
	National Breastfeeding Policy		■	■			
	National Plan for Reducing Maternal and Neonatal Mortality		■	■			
Ministry of Economic and Social Inclusion	Family Care Service Growing with Our Children	■	■	■	■		
	Technical Standard for the Child Development Centre Service	■	■	■	■	■	
	National Plan for the Prevention of Violence Against Children and Adolescents				■		
	Human Development Benefit aimed at Beneficiaries with Minor Children		■		■		
	Benefit for Children in Case of Violent Death of their Mother				■		
	Family Support Modality for Beneficiaries of the Human Development Benefit with Variable Component (BDHV)		■		■		
	Joaquín Gallegos Lara Benefit for People with Disabilities				■		
	“Aliméntate Ecuador” Programme		■		■		
Ministry of Labour	Breastfeeding Support Rooms in the Workplace		■				
	Childcare for Children of Public Servants					■	
	Organic Law on the Right to Human Care					■	
Ministry of Housing	Regulation for Access to Housing Subsidies and Incentives						■
Technical Secretariat	Comprehensive Child Development Operational Manual “Misión Ternura”	■	■	■	■	■	■
	National Strategy Ecuador Grows without Malnutrition	■	■	■	■	■	■
	1000 Days Benefit		■		■		

Note: Policy documents are grouped by lead institution. The table indicates whether each of the six SDH domains (early education, healthcare, health services, social protection, childcare, and housing) was addressed in each policy. A black square (■) denotes explicit policy recommendations within a given domain

Policies led by the health sector (Ministry of Public Health) reflect a strong sectoral focus on health care, access to services, and coverage of biomedical interventions. Health services, understood as systems-level delivery and standards of care, are also commonly addressed but often only in tandem with healthcare itself. In contrast, other determinants received significantly less attention. Housing was the least mentioned and was included only in policies related to malnutrition strategies as a water and sanitation component, and the housing subsidy regulation. Childcare was also underrepresented, typically included only in policies issued by the Ministry of Economic and Social Inclusion or those related to workplace regulations. Early education appeared more frequently in policies associated with integrated child development (e.g. “Misión Ternura”, “Child development centres”) but was rarely mentioned in documents led by the health sector.

Policies led by the Ministry of Economic and Social Inclusion were more likely to address multiple determinants simultaneously in their services, predominantly childcare, health care, and early education. In addition, the determinants of social protection themselves were addressed mainly through targeted interventions such as cash transfer programmes.

Notably, the policies managed by the Technical Secretariat (a recently created ministry-level institution to address chronic child malnutrition) integrate all six social determinants of child health examined, framing them within a strong intersectoral collaboration component and equity-oriented intersectoral package of services focused mainly on prioritised parishes.

To illustrate how current early childhood policies and interventions are connected, we developed a visual map that links their stated objectives with key recommendations (see Supplementary Fig. 2). This figure shows, for instance, how several policies, particularly those from the Ministry of Economic and Social Inclusion (MIES), focus on comprehensive childcare and essential maternal health services. It also highlights the coordinating role of the recent National Strategy Ecuador Grows without Malnutrition, which integrates multiple programmes across the health and social protection sectors.

### Policy document recommendations to promote health equity

For the advance in promoting child health equity, we identified nine policy documents with recommendations related to pure universal policies. It should be noted that we additionally mapped policies using Benach et al.’s (2013) typology of four policy approaches: universal policies with additional focus on gaps (four policy documents), targeted interventions (nine policy documents), redistributive policies (one policy document), and proportionate universalism (six policy documents). Applying this typology framework enables a complementary analysis of whether Ecuadorian early childhood policies move beyond basic universal and targeted interventions to include universal provision with additional intensity for disadvantaged groups, as well as approaches that explicitly aim to redistribute resources or reduce the social gradient in health. Table 4 shows the distribution of equity approaches using the Benach typology and identifies which child population groups are explicitly prioritised in policy texts, with children living in poverty most frequently addressed. It also indicates that seven policy documents adopt an intercultural approach in their document recommendations.

**Table 4:** Policy typologies, intercultural approaches, and focus on vulnerable groups in national early childhood policy recommendations

Lead Institution	Policy Document	Policy Typology	Vulnerable groups	Intercultural approach
Ministry of Public Health	Law on Free Maternity and Child Healthcare	Universal policy with additional focus on gap	Children living in rural or remote areas	
	Procedures for the Healthcare of Prevalent Childhood Illnesses	Universal policy	Children living in poverty, victims of violence, abuse and neglect	
	Zero Malnutrition Project	Targeted Intervention	Children living in poverty	
	Comprehensive Child Healthcare Manual	Universal policy		
	Standard for Essential Obstetric and Neonatal Healthcare	Universal policy		■
	Intersectoral Plan for Food and Nutrition	Proportionate universalism	Children living in poverty, ethnic minorities, children living in rural or remote areas	
	Manual for Monitoring the Nutritional Status of Iodine	Universal policy		
	Manual of Vaccines for Immunopreventable Diseases	Universal policy		
	Manual on the Articulation of Practices and Knowledge of Ancestral Midwives	Proportionate universalism		■
	Neonatal Metabolic Screening Test in Health Units	Universal policy		
	Strategy for the Elimination of Mother-to-Child Transmission of HIV, Syphilis, Hepatitis B, and Chagas	Universal policy		
	Healthcare for Pregnant Women and Neonates in Mobility Conditions	Targeted intervention	Migrants and refugees, children living in rural or remote areas	■
	National Breastfeeding Policy	Universal policy		■
	National Plan for Reducing Maternal and Neonatal Mortality	Universal policy		
Ministry of Economic and Social Inclusion	Family Care Service Growing with our Children	Targeted intervention	Children living in poverty, with disabilities, victims of violence, abuse and neglect, children of a mother in prison	■
	Technical Standards for the Child Development Centre Service	Universal policy with additional focus on gap	Children living in poverty, with disabilities, victims of violence, abuse and neglect, children of a mother in prison	■
	National Plan for the Prevention of Violence against Children and Adolescents	Proportionate universalism	Children living in poverty, ethnic minorities, children with disabilities, migrants and refugees, victims of violence, abuse and neglect	
	Human Development Benefit aimed at Beneficiaries with Minor Children	Targeted intervention	Children living in poverty	
	Benefit for Children in Case of the Violent Death of their Mother	Targeted intervention	Children in orphanhood or single-parent homes	
	Family Support Modality for Beneficiaries of the Human Development Benefit with Variable Component	Targeted intervention	Children living in poverty, with disabilities, victims of violence, abuse and neglect	■
	Joaquín Gallegos Lara Benefit for People with Disabilities	Targeted intervention	Children living in poverty, with disabilities, with catastrophic or rare diseases	
	“Aliméntate Ecuador” Programme	Targeted intervention	Children living in poverty, with disabilities	
Ministry of Labour	Breastfeeding Support Rooms in the Workplace	Universal policy with additional focus on gap		
	Childcare Benefit for Children of Public Servants	Proportionate universalism		
	Organic Law on the Right to Human Care	Universal policy with additional focus on gap		
Ministry of Housing	Regulation Governing Access to Housing Subsidies and Incentives	Redistributive policy	Children living in poverty, ethnic minorities, children with disabilities, children in orphanhood or single-parent homes, with catastrophic or rare diseases, victims of violence, abuse and neglect	
Technical Secretariat	Comprehensive Child Development Operational Manual “Misión Ternura”	Proportionate universalism	Children living in poverty, ethnic minorities, children living in rural or remote areas, victims of violence, abuse and neglect	
	National Strategy Ecuador Grows without Malnutrition	Proportionate universalism	Children living in poverty	
	1000 Days Benefit	Targeted intervention	Children living in poverty	

Note: Policy documents are grouped by lead institution. The table summarises how equity was addressed through recommendations targeting vulnerable groups and classifies policies according to typologies. A black square (■) indicates that the policy recommendation adopted an intercultural approach

Analysing pure universal policies, recommendations for delivering essential universal services reflect a commitment to primary healthcare as the foundation of the national health system. For example, the national vaccination strategy began in 1974 and has become permanent, evolving from a programme focused on maternal and child health to a comprehensive family vaccination initiative. The norms adopted in the early 2000s, such as Procedures for the Healthcare of Prevalent Childhood Illnesses and the Comprehensive Child Healthcare Manual, consolidated primary-level healthcare recommendations to reduce morbidity and mortality in children under five.

For the mother-newborn dyad, the Standards for Essential Obstetric and Neonatal Care and the National Plan for the Reduction of Maternal and Neonatal Mortality emphasise integrated approaches grounded in continuity of health care and prevention. These interventions are framed as essential and have been delivered mainly through first-level health facilities several years ago. Other universal policies have addressed long-standing public health concerns affecting early childhood, including the prevention of iodine deficiency and vertical transmission of infectious diseases such as HIV, syphilis, hepatitis B, and Chagas.

A key broad universal policy is the Breastfeeding National Policy, which emerged to guarantee the effective exercise of the right of young children to breastfeed, as the most suitable means to ensure adequate nutrition. From this policy, numerous strategies have been developed, such as human milk banks, the regulation and control of the promotion of breast milk substitutes in mass media in public and private spaces, and regulations for early bonding and rooming-in.

Concerning policies that allocate prioritised funding and resources to high-risk and disadvantaged populations, the Intersectoral Food and Nutrition Plan, the National Strategy Ecuador Grows Without Malnutrition, and the Comprehensive Child Development Operational Manual “Misión Ternura” are intersectoral policies that exemplify how proportionate universalism policies combine broad accessibility to nutrition and child development services with scaled interventions for the most in-need child population.

In terms of targeted interventions such as cash transfer programmes, the Human Development Benefit (BDH) is the more long-standing policy; it is an extension of the earlier Solidarity Benefit and provides conditional financial support to families with children, contingent on meeting health and education commitments. This programme was later refined through the Benefit for Families with Minor Children, which introduced per-child adjustments. Joaquín Gallegos Lara Benefit is a cash transfer programme created for children with severe disabilities and later reformed to cover children with catastrophic or rare illnesses and those living with HIV/AIDS. More recently, in 2022, the Benefit for Children in Cases of Maternal Violent Death emerged as a response to growing insecurity by addressing new forms of vulnerability in the recent violent context in the country.

In parallel, service-based modalities have expanded under the Ministry of Economic and Social Inclusion, including Growing with our Children, and the Family Support Modality for BDH Beneficiaries, which are targeted interventions focused on children living in economic criticality. Child Development Centres’ policy is building on earlier programmes implemented more than two decades ago. While delivery models, financing arrangements, and providers vary across policy iterations, the policy document consistently defines its core recommendation as providing support to enhance child development to all, but with particular focus on disadvantaged populations. Hence, in this analysis, such policy was classified as universal policies with an additional focus on reducing gaps.

The Regulation Governing Access to Housing Subsidies stands as the sole redistributive policy identified in this analysis. It explicitly prioritises families with vulnerable conditions, such as poverty, ethnic minorities, single-parent households, children with disabilities and others for social housing allocation. By a needs-based allocation mechanism and progressive subsidy structures, the policy actively redistributes resources to reduce housing access inequalities.

For the adoption of an intercultural approach, an illustration of how ethnic communities’ knowledge systems, cultural practices, and worldviews are recognised and integrated in service delivery and policy design is the Manual on the Articulation of Practices and Knowledge of Ancestral Midwives, which formalises the role of ancestral midwifery in institutional childbirth. Other policies as well have acknowledged cultural identity as a determinant of access and trust in early childhood services, promoting health equity through this intercultural perspective.

In summary, while several policy documents include recommendations aimed at addressing the social determinants of health and promoting health equity, their scope, depth, and target populations vary considerably. Some policies presented integrated and multisectoral approaches, whereas others remain sectoral and narrowly focused. These findings highlight both progress and persistent gaps in equity-oriented policy design.

## Discussion

Through the analysis of 29 national-level policy documents, we identified key strengths and constraints in how Ecuadorian policies have addressed SDH and promoted health equity in early childhood. Intersectoral collaboration has been increasingly promoted, yet strategic financing and robust evaluation mechanisms are less systematically embedded across policy design. All six domains of SDH were represented, although unevenly: healthcare and health services received the most attention, whereas housing, early education and childcare received the least. Equity-related approaches have become more prominent by progressively addressing vulnerable groups and integrating intercultural principles, but specific actions targeting ethnic minorities and children with intersecting disadvantages remain inconsistently focused upon. Sectoral variation was also evident: the social protection sector and nutritional policies were more likely to adopt holistic approaches, while early education and housing showed more fragmented or narrowly defined policy contributions.

Our analysis critically assesses the potential of policies to tackle health inequities. While we identified a balance of universal policies and targeted interventions, application of Benach et al.‘s typology exposed a marked deficit: only one redistributive policy (the Regulation Governing Access to Housing Subsidies) was identified. In this regard, evidence reveals that countries with the greatest redistributive policies achieve better early child health and development outcomes and enhance equity [35, 36]. Thus, this policy development gap in structural interventions suggests that current approaches in Ecuador may alleviate inequity superficially without addressing its fundamental determinants.

Despite evidence suggesting policy action on the social determinants of health is essential to advance child health equity, policy mapping studies worldwide have reported variable findings, showing both progress and constraints [37–39]. In Latin America, comparative studies reveal that while most countries have established policy and institutional frameworks supporting early childhood health, notable differences exist in how policies are designed, governed, and implemented [40]. Chile, Mexico, and Peru are often referred to as having more comprehensive early childhood strategies [41, 42] whereas Brazil also stands out for its intersectoral, community-based, large-scale programmes, which include strong components such as parenting support and environmental health [43, 44].

Although there have been remarkable advances in policy design, Latin American countries still face challenges in care provision and equity within fragmented and segmented health systems [19, 45]. Intersectoral strategies are not always accompanied by robust mechanisms for financing and monitoring [46]. Furthermore, most of the Latin American countries still face insufficient operationalisation of intercultural or intersectional approaches, with persistent heterogeneity in implementation and uneven attention to local sociocultural contexts [42, 47, 48].

While no previous systematic policy analysis has been conducted covering a broad range of early childhood policies in Ecuador, existing policy mappings and reports over time have identified recurring challenges, such as fragmented institutional coordination and gaps in implementation and resource allocation [24, 49, 50]. Consistent with our study, these reports also highlighted the lack of financing and robust monitoring and evaluation frameworks [24, 51].

In terms of equity, a recent trend analysis combined with a desk policy review explored ethnic disparities in reproductive, maternal, newborn, and child health between 2004 and 2018. It found considerable improvements in healthcare access for indigenous groups, coinciding with more inclusive legislation and programmes, but persistent inequalities over time underlined the need for stronger monitoring and rigorous impact evaluation [52]. Compared with these focused analyses, our study provides a more comprehensive and cross-sectoral mapping that confirms common patterns observed in Ecuador, while also revealing additional gaps. Despite SDH and equity commitments being increasingly visible in policy design, the operationalisation of equity principles such as intersectionality, territorial differentiation, and specific action for ethnic minorities and disadvantaged groups remains insufficiently attended across the Ecuadorian policy landscape.

### Implications for policy design and governance

Over time, early childhood policies in LMICs increasingly emphasise equity and SDH principles; however, policy design features that enable effective implementation, especially explicit financing arrangements, measurable monitoring frameworks, and clearly allocated intersectoral responsibilities, remain inconsistently specified [53]. Evidence from other settings suggests that even well-aligned intersectoral early childhood policies risk being reduced to conventional sectoral interventions during implementation when governance arrangements, institutional incentives, and delivery capacities are not explicitly addressed at the design stage [54, 55].

Given these gaps, it is timely to reconsider how to address the root causes of health disparities in Ecuador, as well as in other LMICs. First, uneven attention across SDH domains suggests the need for stronger governance structures and intersectoral collaboration. Second, embedding monitoring and evaluation frameworks at the design stage may strengthen accountability, and planning early for sustainability may support continuity of equity-oriented action [56]. Third, more redistributive policies that mobilise revenue and reallocate resources to disadvantaged groups and territories are needed, as highlighted in international policy guidance on equity and sustainable development [36]. Fourth, targeted measures for ethnic minorities and disadvantaged populations require a clearer definition to align with equity principles and community needs; this has broader relevance for LMICs with ethnically diverse populations, where territorial differentiation and community-based planning, with active community engagement, may strengthen equity-oriented actions. Finally, strengthening cross-sectoral data systems can also improve monitoring of service coverage and equity outcomes in early childhood health by adopting a life-course approach, which is consistent with constitutional commitments to intergenerational equity and aligned with policy recommendations at a global scale [57, 58].

Future research should examine how policy intentions are operationalised across the policy cycle, from inception to implementation, and whether services reach the intended beneficiaries across all territories. It is essential to assess how intersectoral collaboration operates in real-world settings and how the intercultural approach is embedded in care delivery. Building on this and given that most policy content centres on health and social protection service delivery in our analysis, it is necessary to evaluate the systems organisation, such as infrastructure, human resources, local planning, and funding, as well as to examine territorial governance as a structural determinant of child health, moving beyond a narrow focus on service outputs.

These insights are relevant to Ecuador and other LMICs facing similar challenges, underscoring the need to strengthen early childhood policy frameworks to transform document commitments effectively into tangible and equitable health outcomes across generations.

## Limitations

This study has some limitations: First, the analysis was based solely on the written content of official policy documents; as such, it identifies priorities and commitments as a reflection of policy agenda rather than proven effectiveness or real impact on health equity. Second, using the six predefined categories from the SDH framework may be restrictive, as our analysis may overlook structural barriers such as political, cultural, commercial, and economic conditions, and may not fully reflect the complexity of systemic factors. Third, some historical documents were unavailable electronically, possibly due to non-publication or archival limitations, a common challenge of document analysis research in LMICs [59]. Thus, while it is true that this study may not capture all extensive early childhood policies from 2000 to 2024, the systematic approach employed provides reasonable confidence that the included policy documents are relevant for addressing the research questions. Finally, since intercoder reliability was not assessed because of the single-coder design, validation followed recommended strategies for solo coding, including a reflexive fieldwork diary, systematic discussions with the research team, and independent advisory board consultation [60–62].

## Conclusion

Like other Latin American countries, Ecuador has made notable progress in designing early childhood policies that address the SDH and aim to advance health equity. However, significant challenges remain in translating policy statements into concrete, equity-related actions. Our results show that critical aspects such as intersectionality, territorial equity, and local adaptation to the needs of disadvantaged groups remain insufficiently incorporated. In addition, we identified only one redistributive policy, suggesting missed opportunities to strengthen structural equity in early years. Consolidating actionable governance structures through stronger cross-sectoral coordination, community-based planning, and articulated strategies for vulnerable groups will be essential to ensure that policies are not merely discursive but also lead to tangible improvements in early childhood health outcomes. Further equity-focused policy evaluation, guided by cross-sectoral disaggregated data and robust health equity indicators, is needed to translate policy into real-world impacts.

## Supplementary Information

Below is the link to the electronic supplementary material.

Supplementary Material 1

Supplementary Material 2

Supplementary Material 3

Supplementary Material 4

Supplementary Material 5

## Data Availability

The textual data analysed in this paper are government policy documents, and no additional data were generated. These policy documents can be found at (http://www.registroficial.gob.ec). The links to access the policy documents (Spanish language) are in Supplementary Table 2.

## Abbreviations

BDH: Bono de Desarrollo Humano (Human Development Benefit)
BDHV: Bono de Desarrollo Humano componente variable (Human Development Benefit with variable component)
CDI: Centros de Desarrollo Infantil (Child Development Centres)
CNH: Creciendo con Nuestros Hijos (Growing with our children)
CONE: Cuidados Obstetricos y Neonatales Esenciales (Essential obstetric and neonatal care)
HIV: Human Immunodeficiency Virus
LMICs: Low- and middle-income countries
MAIS-FCI: Modelo de Atención Integral de Salud Familiar, Comunitario e Intercultural (Comprehensive Family, Community and Intercultural Health Care Model)
MIES: Ministerio de Inclusion económica y social (Social and Economic Inclusion Ministry)
MSP: Ministerio de Salud Publica (Public Health Ministry)
SDGs: Sustainable Development Goals
SDH: Social Determinants of Health
