# Supplementary material for: Addressing social determinants of health and equity in early childhood: a qualitative document analysis of national policies in Ecuador
Source: Int J Equity Health. 2026 May 29;25:180. doi: 10.1186/s12939-026-02891-2 (PMC13422072; doi:10.1186/s12939-026-02891-2)
Supplement: Supplementary file 1 — Supplementary Material 1 [file 12939_2026_2891_MOESM1_ESM.docx]

**
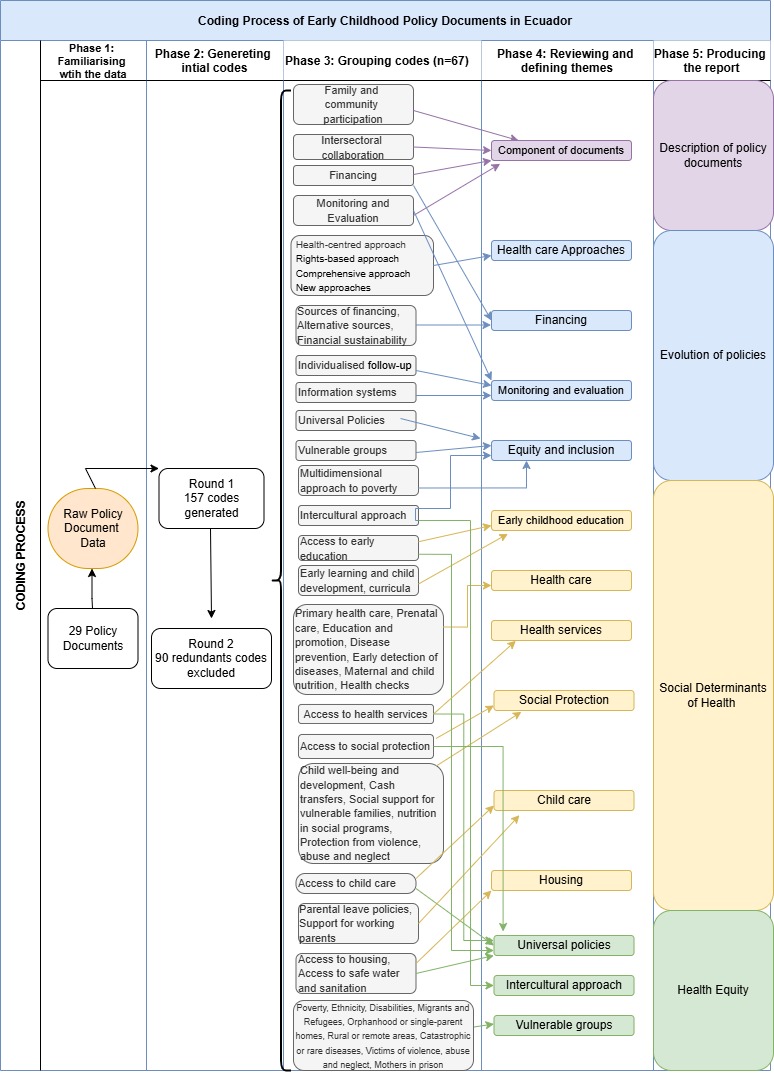
**

**Supplementary Figure 1.** Coding process flow diagram.

Flow diagram illustrating the steps of the coding process used in the qualitative document analysis.
