# Supplementary material for: Addressing social determinants of health and equity in early childhood: a qualitative document analysis of national policies in Ecuador
Source: Int J Equity Health. 2026 May 29;25:180. doi: 10.1186/s12939-026-02891-2 (PMC13422072; doi:10.1186/s12939-026-02891-2)
Supplement: Supplementary file 2 — Supplementary Material 2 [file 12939_2026_2891_MOESM2_ESM.docx]

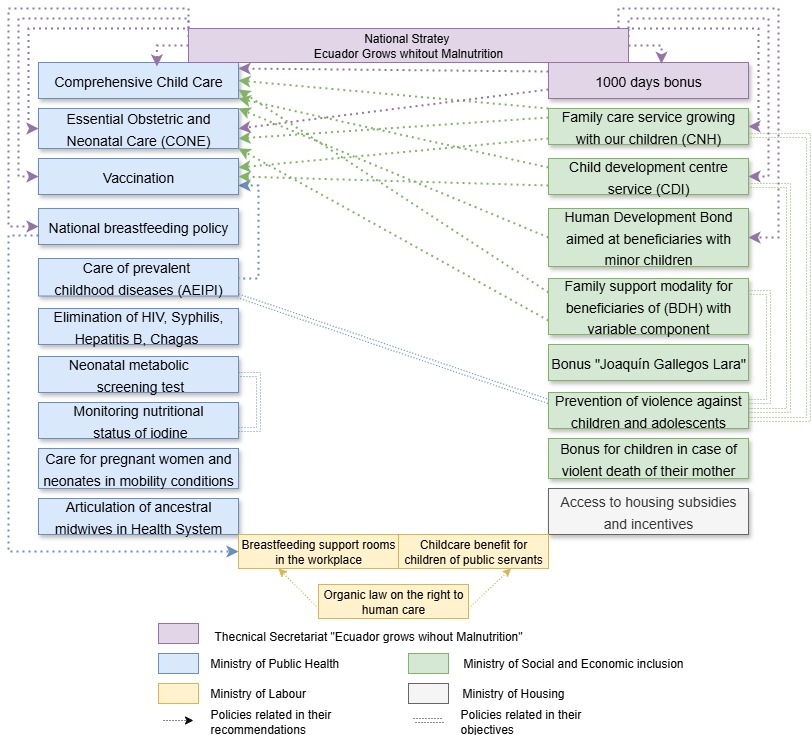


**Supplementary Figure 2.** Early childhood policies in Ecuador (in effect in 2024).

Diagram showing policies in effect in 2024. Double lines indicate shared objectives; dashed arrows represent connections through recommendations. For instance, policies led by the Ministry of Social and Economic Inclusion (green) link mainly to Comprehensive Child Care (including health checks) and Essential Obstetric and Neonatal Care. Importantly, the current National Strategy Ecuador Grows without Malnutrition connects multiple public health policies (e.g. vaccination, breastfeeding, neonatal care) and social protection programmes (e.g. 1000 days bonus, family care services, human development benefit, child development centres).
