# Supplementary material for: Addressing social determinants of health and equity in early childhood: a qualitative document analysis of national policies in Ecuador
Source: Int J Equity Health. 2026 May 29;25:180. doi: 10.1186/s12939-026-02891-2 (PMC13422072; doi:10.1186/s12939-026-02891-2)

**Supplementary Method 1**

**Search strategy**

The primary source was the Lexis (subscription-based legislative repository) that includes official policy instruments such as laws, decrees, ministerial agreements, technical standards, and operational manuals. Lexis repository was authorised for academic research purposes and is referenced here with permission.

The search strategy was conducted in two stages. First, broad open Spanish terms *infantil, infancia, and niñez*, which are commonly used in Ecuadorian policy texts to refer to children and childhood broadly. In the second stage, targeted search terms were applied based on their relevance to the Ecuadorian policy context and their correspondence with key SDH categories.

| **No.** | **Open Search terms (Spanish language)** | **Open Search terms (English language)** | **Results** |
| --- | --- | --- | --- |
| 1 | Infantil | Childhood | 569 |
| 2 | Infancia | Infancy | 219 |
| 3 | Niñez | Children | 864 |
| **No.** | **Category search terms (Spanish language)** | **Category search terms (English language)** | **Results** |
| 1 | educacion inicial | Early education | 167 |
| 2 | sistema de salud* | Health services (health system) | 156 |
| 3 | cuidado infantil | Childcare | 35 |
| 4 | atencion de salud* | Healthcare | 392 |
| 5 | protección social | Social protection | 179 |
| 6 | paternidad/maternidad* | Parenthood | 455 |
| 7 | vivienda (limited "ninos") | Housing | 11 |
|  | TOTAL | | 3047 |

(*) For “healthcare and health services,” both *sistema de salud* and *atención de salud* were included, reflecting how these concepts are articulated in Ecuadorian policy language. “Paternidad/maternidad” was used as a proxy to identify policies related to parental leave, maternity protections, and family support, which are commonly embedded within social protection or employment frameworks.

**Institutional Websites**

| **Institution (Spanish language)** | **Institution (English language)** | **URL** | **Results** |
| --- | --- | --- | --- |
| Ministerio de Desarrollo Urbano y Vivienda(MIDUVI) | Ministry of Urban Development and Housing (MIDUVI) | <https://www.habitatyvivienda.gob.ec/acuerdos_decretos_ministeriales/> | 10 |
| Ministerio de Educación(MINEDUC) | Ministry of Education (MINEDUC) | <https://educacion.gob.ec/documentos-legales-y-normativos/> | 21 |
| Ministerio de Inclusión Económica y Social(MIES) | Ministry of Economic and Social Inclusion (MIES) | <https://biblioteca.inclusion.gob.ec/> | 165 |
| Ministerio de Salud Pública(MSP) | Ministry of Public Health (MSP) | https://www.salud.gob.ec/catalogo-de-normas-politicas-reglamentos-protocolos-manuales-planes-guias-y-otros-del-msp/ | 555 |
|  |  | Total | 751 |

**Grey Literature**

In addition to legislative and institutional sources, a targeted search of grey literature was conducted to identify additional documents referenced in technical reports or programme evaluations. This included reports from international organisations or national observatories. Grey literature was used to complement the main document set by identifying policies that may not be registered in official repositories but were cited or operationalised in the field.

| **Website name/ organisation/**  **author** | **URL** | **Title (Spanish language)** | **Title (English language)** | **Results** |
| --- | --- | --- | --- | --- |
| Comisión Económica para América Latina y el Caribe (CEPAL) | <https://repositorio.cepal.org/bitstreams/6b56efec-d531-45ee-b0cb-6c4ddbcc54e2/download> | Ecuador: reseña de los principales programas sociales y lecciones aprendidas, 2000-2006 | Ecuador: overview of major social programmes and lessons learned, 2000-2006 | 21 |
| Maria Patricia Erazo Ortega, Franklin Orlando Barriga Bedoya | <https://revistainclusiones.org/index.php/inclu/article/view/53> | Políticas para la primera infancia en Ecuador | Early Childhood Policies in Ecuador | 9 |
| César Carranza Barona | <https://biblio.flacsoandes.edu.ec/libros/125475-opac> | Políticas públicas en alimentación y nutrición: Los programas de alimentación social de Ecuador | Public Policies on Food and Nutrition: Ecuador's Social Food Programs | 15 |
| Naciones Unidas, CEPAL, UNICEF | <https://repositorio.cepal.org/bitstream/handle/11362/41233/1/S1700082_es.pdf> | Protección social de la niñez en el Ecuador | Social protection of children in Ecuador | 135 |
| Aldeas infantiles S.O.S | <https://www.aldeasinfantiles.org/getmedia/d59c4650-58fb-400e-b137-86b83decb99a/Mapeo-Politica-Publica-y-Servicios-de-Egreso.pdf> | Mapeo de política pública de sistematización de experiencias de preparación y condiciones para el egreso y atención posterior al egreso del sistema de protección en América Latina y el Caribe | Mapping of public policy for the systematization of experiences of preparation and conditions for discharge and post-discharge care from the protection system in Latin America and the Caribbean | 9 |
| Ministerio de Inclusión Económica y Social, Ministerio de Salud Pública, Unidad de Registro Social | <https://www.inclusion.gob.ec/wp-content/uploads/2023/07/PLAN-DE-PARTICIACION-DE-PARTES-INTERESADAS-1_compressed-1.pdf> | Proyecto Red de Protección Social | Social Protection Network Project | 14 |
|  |  |  | Total | 203 |

**Google Advanced Search**

To supplement the document retrieval process, Google Advanced Search was used with Boolean combinations of Spanish-language keywords. This approach enabled the identification of policy documents that may be hosted on institutional websites but not indexed in formal databases. Only 11 relevant documents retrieved through this method were cross-checked for duplication and assessed against the inclusion criteria.

| **No.** | **Search terms (Spanish language)** | **Search terms (English language)** | **Results** | **Valid results** |
| --- | --- | --- | --- | --- |
| 1 | infantil OR infancia OR ninez | Childhood OR infancy OR children | 2160 | 11 |

Due to the high volume of results, the Google Advanced search was restricted to the following specifications:


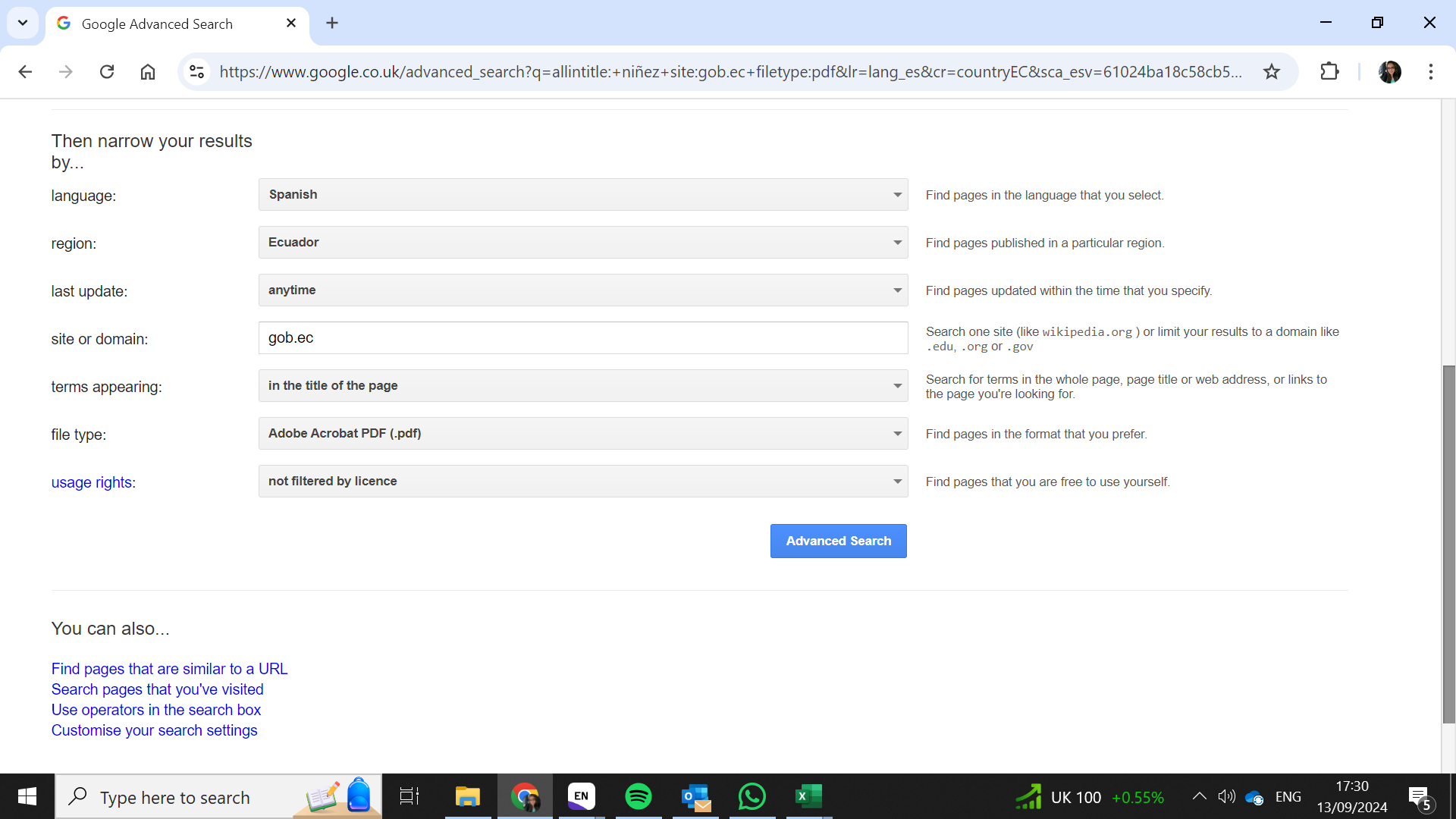

Supplement: Supplementary file 3 — Supplementary Material 3 [file 12939_2026_2891_MOESM3_ESM.docx]
