# Supplementary material for: Addressing social determinants of health and equity in early childhood: a qualitative document analysis of national policies in Ecuador
Source: Int J Equity Health. 2026 May 29;25:180. doi: 10.1186/s12939-026-02891-2 (PMC13422072; doi:10.1186/s12939-026-02891-2)
Supplement: Supplementary file 4 — Supplementary Material 4 [file 12939_2026_2891_MOESM4_ESM.docx]

**Supplementary Table 1.** Coding framework.

| **Themes** | **Codes** | **Code Definition** | **Illustrative excerpt (Spanish)** | **Illustrative excerpt (English)** | **Reference document** |
| --- | --- | --- | --- | --- | --- |
| **Description of components of policy documents** | Family and community participation | Mentions regarding the inclusion of the family and/or community in the creation and/or execution of child health policies. | *“Los CDI deben conformar un comité de familias, mismo que se enfocará en la protección integral de las niñas y niños a través del ejercicio efectivo de sus derechos; la organización de los padres de familia es importante porque requieren establecer acciones de participación activa de las familias y comunidad, que incidan positivamente en el desarrollo integral de las niñas y niños”* | *“The CDI must form a family committee, which will focus on the comprehensive protection of girls and boys through the effective exercise of their rights; the organization of parents is important because it requires establishing actions of active participation of families and the community, which positively impact the comprehensive development of girls and boys.”* | Technical Standard of the CDI Child Development Centre Service, Ministerial Agreement 38 Official Registry Supplement 383 (28 August 2023) |
|  | Intersectoral collaboration | Mention of collaboration between the health, education, and other key sectors to address children's health needs | *“En las Mesas Técnicas Intersectoriales Cantonales, se promueve el compromiso de los diversos actores institucionales, fortaleciendo el liderazgo compartido de los GAD cantonales y las gobernaciones.”* | *In the Cantonal Intersectoral Technical roundtables, the commitment of the various institutional actors is promoted, strengthening the shared leadership of the [local governments].* | Comprehensive Child Development Operational Manual “Mision Ternura”, Ministerial Agreement 3 Official Registry Special Edition 355 (17 February 2020) |
|  | Financing | Mention of mechanisms for the allocation of resources or availability of financing sources to carry out the policy at national and local levels. | *“Aplicación de la metodología de Gestión por Resultados para la asignación de recursos a las instituciones del Estado de acuerdo al cumplimiento de las metas establecidas en el Plan Intersectorial de Alimentación y Nutrición Ecuador”* | *“Application of the Results-Based Management methodology for the allocation of resources to State institutions in accordance with the fulfilment of the goals established in the Intersectoral Food and Nutrition Plan for Ecuador”* | Intersectoral Food and Nutrition Plan, Ministerial Agreement 237, Official Register Special Edition 498 (25 July 2018) |
|  | Monitoring and Evaluation | Mention of the Implementation of systematic and regular processes to evaluate the results of public policies | *Del seguimiento y monitoreo. Las autoridades nacionales de salud, bienestar social, de educación, y de trabajo, realizarán el seguimiento y monitoreo de la implementación de las salas de apoyo a la lactancia y centros de cuidado de desarrollo infantil, de acuerdo al ámbito de sus competencias.* | “Follow-up and monitoring. The national health, social welfare, education, and labour authorities will follow up and monitor the implementation of breastfeeding support rooms and child development care centres, according to the scope of their competences.” | Breastfeeding Support Rooms in Workplaces, Ministerial Agreement 2, Official Register 642 (12 September 2024) |
| **Evolution of Healthcare Approaches** | Health-centred approach | Policies that prioritise the physical and emotional well-being of children, focusing on prevention, medical care and health promotion. | *“Se asegura la necesaria y oportuna atención en los diferentes niveles de complejidad a los recién nacidos o nacidas y sanos o sanas, prematuros-prematuras de bajo peso, y/o con patologías (asfixia perinatal, ictericia, sufrimiento fetal y sepsis), a los niños o niñas menores de 5 años en las enfermedades comprendidas en la estrategia de atención integral de las enfermedades prevalentes de la infancia (AIEPI)”* | *“The necessary and timely care at different levels of complexity is ensured for healthy newborns, premature and low weight infants, and/or with pathologies (perinatal asphyxia, jaundice, foetal distress and sepsis), for children under 5 years of age in the diseases included in the strategy for the integrated management of childhood illnesses (IMCI)”* | Law on Free Maternity and Child Healthcare, Codification 6 Supplement to Official Register 349 (05 September 2006) |
|  |  |  | *“La Estrategia de CONE se enmarca y es parte del Sistema Nacional de Salud, su propósito fundamental es la cobertura y resolución exitosa de los casos de complicaciones obstétricas y neonatales que pongan en riesgo la integridad y la vida de madres y neonatos/as”* | *“The CONE Strategy is framed and is part of the National Health System, its fundamental purpose is the coverage and successful resolution of cases of obstetric and neonatal complications that put the integrity and life of mothers and newborns at risk”* | Standard for Essential Obstetric and Neonatal Care, CONE, Ministerial Agreement 3599 Supplement to the Official Register 39 (18 July 2013) |
|  |  |  | *“Garantizar el diagnóstico, tratamiento y seguimiento oportuno a los neonatos de madres con infección por VIH, sífilis, hepatitis B y enfermedad de Chagas”* | *“Guarantee the timely diagnosis, treatment and follow-up of newborns of mothers with HIV, syphilis, hepatitis B and Chagas disease”* | Strategy for the Elimination of Mother-to-Child Transmission of HIV, Syphilis, Hepatitis B, Chagas, Ministerial Agreement 373, Official Registry Special Edition 1004 (09 July 2019) |
|  | Rights-based approach | Policy approach that prioritises the fundamental rights of children, guaranteeing their access to services and comprehensive protection. | *“Eje 1: Transformación de patrones socioculturales que vulneran el derecho de niñas, niños y adolescentes a una vida libre de violencia.”* | *“Axis 1: Transformation of sociocultural patterns that violate the right of children and adolescents to a life free of violence.”* | National Plan for the Prevention of Violence against Children and Adolescents, Ministerial Agreement 40, Official Register 422 (06 February 2019) |
|  |  |  | *“Las realizaciones se definen como el conjunto de condiciones de vida social, material, de salud, nutrición, juego, aprendizaje y de protección que, en contextos de igualdad de oportunidades, aseguran el desarrollo integral; por tanto, son la expresión y concreción de la garantía y ejercicio de los derechos de la primera infancia.”* | *“Entitlements are defined as the set of conditions of social, material, health, nutrition, play, learning and protection that, in contexts of equal opportunities, ensure comprehensive development; therefore, they are the expression and concretisation of the guarantee and exercise of the rights of early childhood.”* | Family Care Service Growing with our Children, Ministerial Agreement 36, Official Registry Supplement 382 (25 August 2023) |
|  |  |  | *“Esta Ley tiene por objeto tutelar, proteger y regular el derecho al cuidado de personas trabajadoras respecto de sus hijos e hijas, dependientes directos, otros miembros de su familia directa que componen los diferentes tipos de familia, que de manera evidente necesiten su cuidado o protección”* | *“The purpose of this Law is to protect and regulate the right to care of workers with respect to their children, direct dependents, other members of their direct family that make up the different types of family, who clearly need their care or protection.”* | Organic Law on the Right to Human Care, Law 0 Official Registry Supplement 309 (12 May 2023) |
|  | Comprehensive approach | Code that groups policies that seek to improve child well-being in a holistic way, combining education, health, protection and care | *“Fortalecer y generar intervenciones que Incidan sobre los determinantes de la salud, enfocados en la promoción de la salud, la protección social, la seguridad y soberanía alimentaria y agua-saneamiento”* | *“Strengthen and generate interventions that have an impact on the determinants of health, focused on health promotion, social protection, food security and sovereignty and water-sanitation”* | Intersectoral Food and Nutrition Plan, Ministerial Agreement 237 Official Register Special Edition 498 (25 July 2018) |
|  |  |  | *“Las entidades responsables de la provisión y articulación del Paquete Priorizado de bienes y servicios, en el ámbito de sus competencias, son: a) El ente rector de Salud, a través de la Red Pública integral de Salud y Red Privada Complementaria; b) El ente rector de Inclusión Económica y Social, c) La Autoridad Educativa Nacional; y, d) La entidad responsable del Registro Civil, Identificación y Cedulación.”* | *“The entities responsible for the provision and articulation of the Prioritised Package of goods and services, within the scope of their competences, are: a) The governing body of Health, through the Comprehensive Public Health Network and the Complementary Private Network; b) The governing body for Economic and Social Inclusion, c) The National Education Authority; and, d) The entity responsible for the Civil Registry, Identification and Identification.”* | National Strategy Ecuador Grows without Malnutrition, Executive Decree 1211 Supplement 356 of the Official Register (23 December 2020) |
|  |  |  | *“Perfil ideal a alcanzar con la Dimensión: Que todos los Integrantes del núcleo familiar cuenten con un documento de identificación para el acceso a servicios. Que todos los Integrantes del núcleo familiar accedan a consultas de salud preventiva de acuerdo a su edad. Que todos los Integrantes del núcleo familiar que tienen edad entre los 5 y 17 años se encuentren matriculados y asistiendo regularmente a clases y no se encuentren en condiciones de trabajo infantil. Que al menos un integrante de la familia acceda al Crédito de Desarrollo Humano y/o a servicios de empleabilidad. Que la familia establezca un relacionamiento positivo, afianzando la organización de sus integrantes, la distribución equitativa de tareas, para evitar conductas de violencia.”* | *“Ideal profile to be achieved with the Dimension: That all members of the family nucleus have an identification document for access to services. That all members of the family nucleus have access to preventive health consultations according to their age. That all members of the family nucleus who are between 5 and 17 years of age are enrolled and regularly attending classes and are not in conditions of child labour. That at least one member of the family has access to the Human Development Credit and/or employability services. The family establishes a positive relationship, strengthening the organisation of its members, the equitable distribution of tasks, to avoiding violent behaviour.”* | Technical Standard for Family Support Modality for Beneficiaries of the Human Development Benefit with Variable Component (BDHV), Ministerial Agreement 007, Official Registry 250 (14 February 2023) |
|  | New approaches | Incorporation of new models or innovative approaches to improve child health. | *“Enfoque de Género  Implica el análisis de las brechas de desigualdad existentes entre hombres y mujeres, sus relaciones y los roles asignados socialmente de acuerdo al sexo”* | *“Gender Approach involves the analysis of the existing inequality gaps between men and women, their relationships and the roles socially assigned according to sex”* | National Plan for the Prevention of Violence against Children and Adolescents, Ministerial Agreement 40, Official Register 422 (06 February 2019) |
|  |  |  | *“Para el Servicio de Acompañamiento Familiar, el enfoque intergeneracional implica promover en las familias la participación de todos sus integrantes, sin que la edad constituya un factor de exclusión; asegurando que cada persona ejerza sus derechos considerando la etapa en el ciclo de vida en el que se encuentre”* | *“For the Family Accompaniment Service, the intergenerational approach implies promoting the participation of all its members in families, without age being an exclusion factor; ensuring that each person exercises their rights considering the stage in the life cycle in which they find themselves”* | Technical Standard for Family Support Modality for Beneficiaries of the Human Development Benefit with Variable Component (BDHV), Ministerial Agreement 007, Official Registry 250 (14 February 2023) |
|  |  |  | *“Enfoque de prevención de violencia.  Los servicios de desarrollo infantil integral deben convertirse en entornos protectores libres de violencia, generando acuerdos que permitan identificar, intervenir y referir posibles casos de violencia contra niñas, niños y demás miembros de la familia, en cumplimiento de sus derechos y respeto a las diferencias”* | *“Violence prevention approach.  Comprehensive child development services must become protective environments free of violence, generating agreements that make it possible to identify, intervene and refer possible cases of violence against children and other family members, in compliance with their rights and respect for differences”* | Technical Standard of the CDI Child Development Centre Service, Ministerial Agreement 38 Official Registry Supplement 383 (28 August 2023) |
| **Evolution of Financing** | Sources of financing | Mention of mechanisms for the allocation of resources or availability of financing sources to carry out the policy at the national and local levels. | *“El bono se entregará a través de la malla presupuestaria que administra la Vicepresidencia de la República en coordinación con el Ministerio de Finanzas, quien instrumentará la herramienta informática necesaria.”* | *“The Benefit will be delivered through the budget administered by the Vice Presidency of the Republic in coordination with the Ministry of Finance, which will implement the necessary IT tool.”* | Creation of Joaquín Gallegos Lara Benefit in favour of people with disabilities, Executive Decree 422 Supplement Official Registry 252 (06 August 2010) |
|  |  |  | *“Las inclusiones mensuales y la población total cubierta por esta transferencia monetaria, se encontrará condicionada a la asignación presupuestaria institucional realizada por el ente rector de las finanzas públicas para un ejercicio fiscal, en función de los recursos disponibles”* | *“The monthly inclusions and the total population covered by this cash transfer will be based on institutional budget allocation made by the governing body of public finances for a fiscal year, subject to available resources”* | 1000-day Benefit, Executive Decree 435 Official Registry Supplement 84 (15 June 2022) |
|  |  |  | *“La aplicación por parte de las entidades públicas que cumplan las condiciones estipuladas en el presente Acuerdo será financiado con cargo a las asignaciones presupuestarias institucionales, toda vez que el Ministerio de Economía y Finanzas no asumirá ninguna obligación, ni comprometerá recursos financieros adicionales del Presupuesto General del Estado para dicho efecto.”* | *“The application by public entities that meet the conditions stipulated in this Agreement will be financed from institutional budgetary allocations, since the Ministry of Economy and Finance will not assume any obligation, nor will it commit additional financial resources from the General State Budget for that purpose.”* | Childcare Benefit for Children of Public Servants, Ministerial Agreement 85 Supplement to Official Registry 344 (03 July 2023) |
|  | Alternative sources | Identification and use of financing mechanisms outside the traditional state budget to support early childhood policies. | *“Los fondos solidarios locales de salud, quedan en la libertad de agregar prestaciones de salud, requeridas por el análisis epidemiológico de la Dirección Provincial de Salud y socio-económico local, en el marco que determine el Sistema Nacional de Salud, identificando fuentes adicionales de financiamiento”* | *“The local solidarity health funds are free to add health benefits, required by the epidemiological analysis of the Provincial Directorate of Health and local socio-economic, within the framework determined by the National Health System, identifying additional sources of financing”* | Law on Free Maternity and Child Healthcare, Codification 6 Supplement to Official Register 349 (05 September 2006) |
|  |  |  | *“Créase en cada municipio, los Fondos Solidarios Locales de Salud, que recibirán los recursos financieros del Fondo Solidario de Salud, para garantizar la aplicación de la ley.”* | *“Local Solidarity Health Funds shall be created in each municipality, which will receive financial resources from the Solidarity Health Fund, to ensure the application of the law.”* | Law on Free Maternity and Child Healthcare, Codification 6 Supplement to Official Register 349 (05 September 2006) |
|  |  |  | *“En 2015, se firma el Convenio de cooperación entre MSP y OPS, en el cual se establece que todas las vacunas, jeringas, implementos y equipos de cadena de frío serán adquiridos a través del Fondo Rotatorio, encontrándose respaldado por la Ley Orgánica de Salud”* | *“In 2015, the Cooperation Agreement between MSP and PAHO was signed, in which it is established that all vaccines, syringes, implements and cold chain equipment will be acquired through the Revolving Fund, being supported by the Organic Law of Health”* | Manual of Vaccines for Immunopreventable Diseases, Ministerial Agreement 63, Official Registry Special Edition 143 (28 November 2019) |
|  | Financial sustainability | Ensure that financial resources allocated to early childhood policies are sufficient and sustainable in the long term, ensuring the continuity of the programmes. | *“El ente rector de las Finanzas Públicas será responsable de la asignación presupuestaria del “paquete priorizado” establecido en el artículo 2 del presente Decreto. La asignación presupuestaria vigente, para estos servicios, no podrá ser objeto de reducción”* | *“The governing body of Public Finances will be responsible for the budgetary allocation of the “prioritised package” established in Article 2 of this Decree. The current budget allocation for these services may not be subject to reduction”* | National Strategy Ecuador Grows without Malnutrition, Executive Decree 1211 Supplement 356 of the Official Register (23 December 2020) |
| **Evolution of Monitoring and evaluation** | Monitoring and evaluation | Implementation of systematic and regular processes to evaluate the results of public policies | *“La medición se realizará según el indicador en los periodos de tiempo: mensual, trimestral, semestral, anual y al término.”* | *“The measurement will be carried out according to the indicator in the periods: monthly, quarterly, semi-annually, annually and at the end.”* | National Plan for the Reduction of Maternal and Neonatal Mortality, Ministerial Agreement 60 (20 August 2008) |
|  |  |  | *“El Monitoreo y Evaluación para la implementación de las Redes CONE en el País, permitirá medir el impacto del comportamiento y cobertura de la atención en todos los niveles CONE.”* | *“The Monitoring and Evaluation for the implementation of the CONE Networks in the country will make it possible to measure the impact of behaviour and coverage of care at all CONE levels”* | Standard for Essential Obstetric and Neonatal Care, CONE, Ministerial Agreement 3599 Supplement to the Official Register 39 (18 July 2013) |
|  |  |  | *“Este padrón, en tanto prestaciones permite el seguimiento y monitoreo del paquete integral e intersectorial de servicios; y en tanto trayectoria de niños y niñas, permite el acompañamiento nominal del paquete completo de desarrollo infantil (peso, talla, hemoglobina, control de crecimiento, logros de aprendizaje).”* | *“This registry, as benefits, allows the follow-up and monitoring of the comprehensive and intersectoral package of services; and as a trajectory of boys and girls, it allows the individual accompaniment of the complete package of child development (weight, height, haemoglobin, growth control, learning achievements).”* | Comprehensive Child Development Operational Manual “Mision Ternura”, Ministerial Agreement 3, Official Registry Special Edition 355 (17 February 2020) |
|  |  |  | *“Los CDI deben cumplir con el registro, verificación y gestión de las atenciones intersectoriales recibidas por las niñas y niños, lo que permitirá tener un seguimiento del cumplimiento de sus derechos como el resultado de la aplicación de indicadores de desarrollo, información del seguimiento individualizado al control salud, nutrición y otros; el ingreso de esta información se debe realizar en el sistema informático vigente”* | *“The CDIs must comply with the registration, verification and management of the intersectoral care received by children, which will allow them to monitor the fulfilment of their rights as a result of the application of development indicators, information on individualised follow-up to health, nutrition and other controls; The entry of this information must be made in the current computer system”* | Technical Standard of the CDI Child Development Centre Service, Ministerial Agreement 38, Official Registry Supplement 383 (28 August 2023) |
|  | Individualised follow-up | An approach that focuses on personalised monitoring of each child or family to ensure that their specific needs are met and results are achieved. | *“Diseñar e implementar un sistema de registro único nominal de atención y seguimiento a niñas, niños y adolescentes víctimas de violencia y emitir protocolos para el uso de los registros administrativos y el registro único”* | *“Design and implement a system of single nominal registry of care and follow-up for children and adolescents, victims of violence and issue protocols for the use of administrative records and the single registry”* | National Plan for the Prevention of Violence against Children and Adolescents, Ministerial Agreement 40, Official Register 422 (06 February 2019) |
|  |  |  | *“El Seguimiento Nominal será el mecanismo mediante el cual se realizará el control a la prestación del Paquete Priorizado, a través del cual se verificará de forma intersectorial la entrega oportuna de las prestaciones que recibe la población objetivo de la presente Estrategia, garantizando su atención integral”* | *“Individual Monitoring will be the mechanism through which control will be carried out on the provision of the Prioritised Package, through which the timely delivery of the benefits received by the target population of this Strategy will be verified in an intersectoral manner, guaranteeing their comprehensive care.”* | National Strategy Ecuador Grows without Malnutrition, Executive Decree 1211 Supplement 356 of the Official Register (23 December 2020) |
|  |  |  | *“La coordinadora/coordinador técnico territorial del MIES o quien haga las veces en la institución pública-privada con el apoyo de la educadora o educador familiar debe realizar el seguimiento nominal de la trayectoria de vida de cada niña, niño y mujer gestante, a todas las atenciones brindadas que permitan alcanzar sus realizaciones de manera efectiva y en el momento oportuno”* | *“The territorial technical coordinator of the MIES or whoever takes the place of the public-private institution, with the support of the family educator, must carry out the individual monitoring of the life trajectory of each child and pregnant woman, to all the care provided that allows them to achieve their entitlements effectively and at the right time”* | Family Care Service Growing with our Children, Ministerial Agreement 36 Official Registry Supplement 382 (25 August 2023) |
|  | Information systems | Mention of the Development and implementation of databases and technological systems that allow the collection and analysis of data to evaluate the impact of early childhood policies or to carry out epidemiological surveillance | *Para el “Seguimiento Nominal” de las prestaciones que recibe la población objetivo, las entidades responsables del “Paquete Priorizado”, bajo la coordinación y en conjunto con la Secretaría Técnica Ecuador Crece Sin Desnutrición Infantil, implementarán una plataforma intersectorial de corte longitudinal con interoperabilidad de datos, con su respectivo modelo de gestión, con la finalidad de garantizar la entrega periódica, oportuna y depurada de sus padrones nominales y/o bases de datos nominales, únicos, identificables e interoperables, salvaguardando la confidencialidad de estos……..”* | *For the “Individual Monitoring” of the benefits received by the target population, the entities responsible for the “Prioritised Package”, under the coordination and in conjunction with the Technical Secretariat Ecuador Grows Without Child Malnutrition, will implement a longitudinal intersectoral platform with data interoperability, with their respective management model, in order to guarantee periodic delivery, timely and purified of their nominal registers and/or nominal, unique, identifiable and interoperable databases, safeguarding their confidentiality…”* | National Strategy Ecuador Grows without Malnutrition, Executive Decree 1211 Supplement 356 of the Official Register (23 December 2020) |
|  |  |  | *“Seguimiento Nominal.  Es un sistema informático de seguimiento y padrón nominal implementado por el MIES; es un mecanismo a través del cual se realiza el monitoreo de las prestaciones públicas que recibe un mismo individuo a lo largo del ciclo de vida.”* | *“Individual Monitoring.  It is a computerised system of monitoring and nominal registry implemented by the MIES; it is a mechanism through which the monitoring of the public benefits received by the same individual throughout the life cycle is carried out.”* | Technical Standard of the CDI Child Development Centre Service, Ministerial Agreement 38 Official Registry Supplement 383 (28 August 2023) |
|  |  |  | *“La coordinadora/coordinador técnico territorial del MIES o quien haga las veces en la institución pública-privada con el apoyo de la educadora o educador familiar debe realizar el seguimiento nominal de la trayectoria de vida de cada niña, niño y mujer gestante, a todas las atenciones brindadas que permitan alcanzar sus realizaciones de manera efectiva y en el momento oportuno.”* | *“The territorial technical coordinator of the MIES or whoever takes the place of the public-private institution with the support of the family educator must carry out the nominal monitoring of the life trajectory of each child and pregnant woman, to all the care provided that allows them to achieve their achievements effectively and at the right time.”* | Family Care Service Growing with our Children, Ministerial Agreement 36, Official Registry Supplement 382 (25 August 2023) |
| **Evolution of equity and social inclusion** | Universal Policies | Strategies and programmes designed to benefit all children without distinction, guaranteeing their access to basic services such as health, education and nutrition. | *“Disponer que la prestación del servicio de Tamizaje Metabólico Neonatal se realice en forma obligatoria a todo niño /a al nacer, en todos los establecimientos de salud que conforman el Sistema Nacional de Salud”* | *“To provide that the provision of the Neonatal Metabolic Screening service is mandatory for all children at birth, in all health facilities that make up the National Health System”* | Neonatal Metabolic Screening Test in Health Units, Ministerial Agreement 4779, Official Register 222 (09 April 2014) |
|  |  |  | *“La Autoridad Educativa Nacional, garantizará el acceso y permanencia en un entorno seguro de las gestantes en el Sistema Nacional de Educación articulando su atención en el sistema de salud, así como la provisión del “Paquete Priorizado “ a sus hijos e hijas, en el ámbito de las competencias de cada Cartera de Estado, debiendo para el efecto, reportar los indicadores de proceso y los resultados”* | *“The National Education Authority will guarantee access to and permanence in a safe environment for pregnant women in the National Education System, articulating their care in the health system, as well as the provision of the “Prioritised Package” to their sons and daughters, within the scope of the competences of each State Portfolio, and for this purpose, report process indicators and results”* | National Strategy Ecuador Grows without Malnutrition, Executive Decree 1211 Supplement 356 of the Official Register (23 December 2020) |
|  |  |  | *“La educadora/educador familiar del MIES debe informar oportunamente a todas las familias de las niñas y niños (incluidos quienes están con condición de discapacidad) que cumplen la edad establecida por el Ministerio de Educación - MINEDUC, sobre el proceso para ingresar al sistema educativo y del egreso que se realizará del servicio CNH una vez que el MINEDUC inicie las clases.”* | *“The MIES family educator must inform all families of children (including those with disabilities) who reach the age established by the Ministry of Education - MINEDUC, in a timely manner, about the process to enter the educational system and the exit that will be made from the CNH service once the MINEDUC starts classes.”* | Family Care Service Growing with our Children, Ministerial Agreement 36 Official Registry Supplement 382 (25 August 2023) |
|  | Vulnerable groups | Policies and programmes that focus their efforts on children and families in the most vulnerable situations. | *“Se crea el Bono “Joaquín Gallegos Lara” a favor de las personas con discapacidad severa y profunda en situación crítica que no pueden gobernarse por si mismos identificadas como tales en la base de datos de la ''Misión Solidaria Manuela Espejo”, con el propósito de contribuir a mejorar sus condiciones de vida”* | *“The “Joaquín Gallegos Lara” Benefit is created in favor of people with severe and profound disabilities in critical situations who cannot govern themselves identified as such in the database of the ''Manuela Espejo Solidarity Mission'', with the purpose of contributing to improving their living conditions.* | Creates Joaquín Gallegos Lara Benefit in favour of people with disabilities, Executive Decree 422 Supplement Official Registry 252 (06 August 2010) |
|  |  |  | *“Este lineamiento estratégico plantea la voluntad del Estado por potenciar las intervenciones en materia de inclusión, que estén vinculadas a los determinantes de una buena alimentación y nutrición, con énfasis en los grupos de atención prioritaria y la población que se encuentra en pobreza o vulnerabilidad”* | *“This strategic guideline sets out the State's willingness to promote interventions in the area of inclusion, which are linked to the determinants of good food and nutrition, with emphasis on priority attention groups and the population that is in poverty or vulnerability”* | Intersectoral Food and Nutrition Plan, Ministerial Agreement 237 Official Register Special Edition 498 (25 July 2018) |
|  |  |  | *“Investigaciones sobre violencia contra niñas, niños y adolescentes considerando sus especiﬁcidades: discapacidad, autoidentiﬁcación étnica, población LGBTI y residencia en zonas de frontera.”* | *“Research on violence against children and adolescents considering their specificities: disability, ethnic self-identification, LGBTI population and residence in border areas.”* | National Plan for the Prevention of Violence against Children and Adolescents, Ministerial Agreement 40, Official Register 422 (06 February 2019) |
|  | Multidimensional approach to poverty | An approach that not only addresses economic poverty, but also other related factors such as access to education, adequate housing, health and nutrition. | *“Esta transferencia monetaria busca garantizar un piso mínimo de consumo, así como incentivar el uso de servicios de salud y de desarrollo infantil, y la sensibilización sobre la nutrición materna infantil y buenas prácticas de salud a través de la complementariedad con los servicios de desarrollo infantil integral.”* | *“This cash transfer seeks to guarantee a minimum consumption floor, as well as to encourage the use of health and child development services, and to raise awareness about maternal and child nutrition and good health practices through complementarity with comprehensive child development services.”* | 1000-day Benefit, Executive Decree 435, Official Registry Supplement 84 (15 June 2022) |
|  |  |  | *“El Servicio tiene su base en torno a cinco (5) dimensiones que integran catorce (14) condiciones básicas de desarrollo familiar (CBDF) y catorce (14) indicadores definidos. Las dimensiones ofrecen una metodología para trabajar con las familias y fomentan la gestión de procesos por el lado de la oferta del servicio, incluyendo el trabajo de coordinación interinstitucional e intersectorial, fundamental para el cumplimiento de la corresponsabilidad”* | *“The Service is based on around five (5) dimensions that integrate fourteen (14) basic conditions of family development (CBDF) and fourteen (14) defined indicators. The dimensions offer a methodology for working with families and promote the management of processes on the supply side of the service, including inter-institutional and intersectoral coordination work, which is essential for the fulfilment of co-responsibility.”* | Technical Standard for Family Support Modality for Beneficiaries of the Human Development Benefit with Variable Component (BDHV), Ministerial Agreement 007, Official Registry 250 (14 February 2023) |
|  | Intercultural approach | Incorporating cultural elements into early childhood policies, recognising and respecting cultural diversity and adapting services to the needs of different communities and ethnic groups. | *“Por lo tanto, es importante incorporar en CONE el enfoque holístico del sumak kawsay y operativizar el enfoque intercultural en los tres niveles de atención, mediante acciones de: a) formación y capacitación del talento humano en competencias interculturales de gestión y atención; b) aplicación de estrategias y herramientas de interacción con el paciente en función de las diferencias culturales y de sus prácticas en salud; y c) fortalecimiento de la pertinencia cultural en la prestación de cuidados obstétricos y neonatales esenciales y de emergencia.”* | *“Therefore, it is important to incorporate the holistic approach of sumak kawsay into CONE and to operationalise the intercultural approach at the three levels of care, through actions of: a) training and training of human talent in intercultural management and care competencies; b) application of strategies and tools for interaction with patients based on cultural differences and their health practices; and (c) strengthening cultural relevance in the provision of essential and emergency obstetric and neonatal care.”* | Standard for Essential Obstetric and Neonatal Care, CONE, Ministerial Agreement 3599 Supplement to the Official Register 39 (18 July 2013) |
|  |  |  | *“La atención del parto institucional por parteras y parteros ancestrales estará sujeta a prácticas beneficiosas de la partería ancestral: uso de plantas medicinales, técnicas ancestrales, parto en libre posición, apego inmediato, pinzamiento oportuno del cordón umbilical, lactancia en la primera hora de vida, entre otras”* | *“Institutional childbirth care by ancestral midwives will be subject to beneficial practices of ancestral midwifery: use of medicinal plants, ancestral techniques, free-position childbirth, immediate attachment, timely clamping of the umbilical cord, breastfeeding in the first hour of life, among others”* | Manual on the Articulation of Practices and Knowledge of Ancestral Midwives, Ministerial Agreement 161 Official Register 347 (06 July 2023) |
|  |  |  | *“El personal de salud deberá brindar atención de forma prioritaria, sin discriminación alguna y tomando en cuenta todos los conceptos de movilidad humana y respetar la interculturalidad de la paciente acorde a su origen y creencia.”* | *“Health personnel must provide care as a priority, without any discrimination and taking into account all concepts of human mobility and respect the interculturality of the patient according to her origin and belief.”* | Healthcare of Pregnant Women and Newborns in Mobility Conditions, Ministerial Agreement 98, Official Registry Supplement 589 (28 June 2024) |
| **Early childhood education** | Access to education | Recommendations that promote access to early childhood education for the entire target population without discrimination | *“Según lo establecido en la Política Pública de la Primera Infancia, el vínculo de las niños y niños en los servicios de desarrollo infantil, culmina una vez que cumplen los 36 meses de edad, luego de lo cual, se promueve su continuidad hacia los programas del Ministerio de Educación, de acuerdo a lo dispuesto en las Normas Técnicas CDI y CNH”* | *“According to the provisions of the Early Childhood Public Policy, the link between children and child development services ends once they reach 36 months of age, after which their continuity is promoted towards the Ministry of Education programmes, in accordance with the provisions of the CDI and CNH Technical Standards.”* | Comprehensive Child Development Operational Manual “Mision Ternura”, Ministerial Agreement 3, Official Registry Special Edition 355 (17 February 2020) |
|  | Early learning and child development | Recommendations for early childhood education and care services to focus on children's cognitive, emotional and social development. | *“El Currículo de Educación Inicial del MINEDUC vigente, plantea cuatro ámbitos de desarrollo y aprendizaje, destrezas, así como objetivos de aprendizaje, específicamente para las niñas y niños menores de 3 años de edad. Estos objetivos orientan las experiencias de aprendizaje que requieren las niñas y niños... posibilitando logros de aprendizaje en cada edad”* | *“The current MINEDUC Early Childhood Education Curriculum proposes four areas of development and learning, skills, as well as learning objectives, specifically for girls and boys under 3 years of age. These objectives guide the learning experiences that girls and boys require... enabling learning achievements at each age.”* | Technical Standard of the CDI Child Development Centre Service, Ministerial Agreement 38, Official Registry Supplement 383 (28 August 2023) |
|  | Education curricula | Recommendations for educational guides and content designed for the education of children in the early years of life. | *“El Currículo de Educación Inicial del MINEDUC vigente, plantea cuatro ámbitos de desarrollo y aprendizaje, destrezas, así como objetivos de aprendizaje, específicamente para las niñas y niños menores de 3 años de edad.”* | *“The current MINEDUC Early Childhood Education Curricula proposes four areas of development and learning, skills, as well as learning objectives, specifically for girls and boys under 3 years of age.”* | Technical Standard of the CDI Child Development Centre Service, Ministerial Agreement 38, Official Registry Supplement 383 (28 August 2023) |
| **Health care** | Primary health care | Recommendations for providing essential health services that cover the needs for prevention, diagnosis, treatment and monitoring of common diseases, especially at the community level. | *“Nivel CONE Comunitario.... se define como un Modelo local de organización y coordinación para la atención Materno Neonatal que es liderada por los proveedores de servicios de salud institucionales del Primer Nivel de Atención del MSP con la participación de los proveedores de salud comunitarios (Parteras Tradicionales) en una Parroquia o Circuito de Salud, teniendo como fin incrementar el acceso de la población más vulnerable, garantizando la atención de CONE continua y de calidad a la mayor cantidad de usuarias/os”* | *“Community CONE Level... is defined as a local model of organisation and coordination for Maternal Neonatal care that is led by institutional health service providers of the First Level of Care of the MSP with the participation of community health providers (Traditional Midwives) in a Parish or Health Circuit, to increase access for the most vulnerable population, guaranteeing continuous and quality CONE care to the greatest number of users.”* | Standard for Essential Obstetric and Neonatal Care, CONE, Ministerial Agreement 3599 Supplement to the Official Register 39 (18 July 2013) |
|  | Prenatal care | Recommendations for addressing maternal health during pregnancy and its relationship to child health. | *“Implementar las normas y protocolos interculturales de anticoncepción, de atención materno-neonatal, sistema de vigilancia epidemiológica y monitoreo de estándares/indicadores para la mejora continua de la calidad de atención materna y neonatal”* | *“Implement intercultural standards and protocols for contraception, maternal-neonatal care, epidemiological surveillance system and monitoring of standards/indicators for continuous improvement of the quality of maternal and neonatal care”* | National Plan for the Reduction of Maternal and Neonatal Mortality, Ministerial Agreement 474 (20 August 2008) |
|  | Education and promotion of children's health | Recommendations aimed at promoting healthy habits, disease prevention and the comprehensive well-being of children through education and health promotion actions for parents, family and community | *“El ente rector de Inclusión Económica y Social en coordinación con el ente rector de Salud.......brindará sesiones de educación familiar prenatal y neonatal, que contemple, entre otros, lavado de manos, higiene alimentaria, lactancia materna y consumo de agua segura; verificando que la población de los servicios Creciendo con Nuestros Hijos y Centros de Desarrollo Infantil sea beneficiaria....”* | *“The governing body of Economic and Social Inclusion in coordination with the governing body of Health... will provide prenatal and neonatal family education sessions, which include, among others, hand washing, food hygiene, breastfeeding and safe water consumption; verifying that the population of the Growing with Our Children services and Child Development centres are beneficiaries....”* | National Strategy Ecuador Grows without Malnutrition, Executive Decree 1211 Supplement 356 of the Official Register (23 December 2020) |
|  | Disease prevention | Recommendations to strengthen disease prevention in early childhood | *“Todo tipo de sal alimentaria, usada en el hogar y en los alimentos procesados, debe estar fortificada con yodo como una estrategia segura y efectiva para la prevención y control de los desórdenes por deficiencia de yodo, en poblaciones viviendo en condiciones estables o en situación de emergencia”* | *“All types of dietary salt, used in the home and in processed foods, should be fortified with iodine as a safe and effective strategy for the prevention and control of iodine deficiency disorders, in populations living in stable conditions or in emergencies”* | Manual for Monitoring Nutritional Status of Iodine, Ministerial Agreement 120, Official Register 310 (15 May 2023) |
|  | Early detection of diseases | Strategies to improve the early detection of diseases as primary prevention and screening strategies | *“Implementar la prueba del Tamizaje Metabólico Neonatal en todas las unidades de salud que conforman el Sistema Nacional de Salud, con el fin de determinar y prevenir discapacidades presentes o que pudieren desarrollarse en los recién nacidos vivos en el territorio ecuatoriano”* | *“Implement the Neonatal Metabolic Screening test in all health units that make up the National Health System, in order to determine and prevent disabilities present or that could develop in live newborns in Ecuadorian territory”* | Neonatal Metabolic Screening Test in Health Units, Ministerial Agreement 4779, Official Register 222 (09 April 2014) |
|  | Maternal and child nutrition | Recommendations to promote adequate nutrition for mothers during pregnancy and breastfeeding, as well as optimal nutrition for children from birth to the first years of life | *“Componente técnico de atención.- Consiste en mejorar la nutrición materna de las embarazadas adultas y adolescentes; eliminar de manera progresiva la desnutrición en los niños menores de un año, especialmente la desnutrición crónica; eliminar de manera progresiva las deficiencias nutricionales, especialmente la anemia por falta de hierro; prevenir y controlar el sobrepeso y la obesidad”* | *“Technical care component.- It consists of improving maternal nutrition in pregnant women, adults and adolescents; progressively eliminating malnutrition in children under one year of age, especially chronic malnutrition; progressively eliminating nutritional deficiencies, especially anemia due to lack of iron; preventing and controlling overweight and obesity”* | Creation of Zero Malnutrition Project, Ministerial Agreement 175, Official Register 411 (23 March 2011) |
|  | Health checks | Recommendations to ensure that children receive regular medical check-ups | *“Coordinar con la o las unidades del MSP de los sectores asignados, el cumplimiento de los controles de salud a mujeres gestantes, niñas y niños de 0 a 3 años, según la normativa vigente; y, apoyar en encuentros familiares para el fortalecimiento en los temas de salud y nutrición”* | *“Coordinate with the MSP unit(s) of the assigned sectors, the fulfilment of health controls for pregnant women, girls and boys from 0 to 3 years old, according to current regulations; and, support family meetings to strengthen health and nutrition issues”* | Family Care Service Growing with our Children, Ministerial Agreement 36 Official Registry Supplement 382 (25 08 2023) |
| **Health services** | Access to health services | Recommendations aimed at ensuring that all children have coverage and access to health services without discrimination. | *“Toda mujer tiene derecho a la atención de salud gratuita y de calidad durante su embarazo, parto y postparto, así como al acceso a programas de salud sexual y reproductiva. De igual manera se otorgará sin costo la atención de salud a los recién nacidos-nacidas y niños-niñas menores de cinco años, como una acción de salud pública, responsabilidad del Estado”* | *“Every woman has the right to free, quality health care during pregnancy, childbirth and postpartum, as well as access to sexual and reproductive health programmes. Likewise, health care will be provided free of charge to newborns and children under five years of age, as a public health action, the responsibility of the State.”* | Law on Free Maternity and Child Healthcare, Codification 6 Supplement to Official Register 349 (09 May 2006) |
|  | Quality of health services | Recommendations to ensure the provision of quality health services. | *“Cada establecimiento de salud del Primer, Segundo y Tercer Nivel de Atención debe contar con un Equipo de Mejoramiento Continuo de la Calidad (EMCC) que es el responsable directo de las intervenciones técnicas para mejorar la calidad en la atención materna neonatal con pertinencia cultural y enfoque de derechos humanos”* | *“Each health facility at the First, Second and Third Level of Care must have a Continuous Quality Improvement Team (EMCC) that is directly responsible for technical interventions to improve the quality of maternal neonatal care with cultural relevance and a human rights approach”* | Standard for Essential Obstetric and Neonatal Care, CONE, Ministerial Agreement 3599 Supplement to the Official Register 39 (18 July 2013) |
| **Social protection** | Access to social protection | Recommendations aimed at ensuring that all children have coverage and access to child protection services without discrimination. | *“Los Servicios de Desarrollo Infantil CDI Públicos (directos - convenios) brindaran atención a niñas y niños de 1 a 3 años de edad; los CDI Privados y Fiscomisionales aseguran la atención a niñas y niños entre 45 días de nacidos a 3 años de edad, con acceso, cobertura y calidad en los servicios a través de la jornada diaria con actividades de juego y aprendizaje, acciones de salud y nutrición, entornos protectores, promoviendo la corresponsabilidad de la familia y la comunidad”* | *“Public Child Development Services (CDI) (direct - agreements) will provide care to girls and boys from 1 to 3 years of age; Private and Fiscomisional CDIs ensure care for girls and boys between 45 days old and 3 years of age, with access, coverage and quality in services throughout the daily schedule with play and learning activities, health and nutrition actions, protective environments, promoting the co-responsibility of the family and the community”* | Technical Standard of the CDI Child Development Centre Service, Ministerial Agreement 38, Official Registry Supplement 383 (28 August 2023) |
|  | Child well-being and development | Recommendations that guarantee healthy growth, comprehensive protection and optimal development of children in their early stages of life. | *“Promover la protección integral en la prestación de los servicios de desarrollo infantil integral para la primera infancia, que incluye a centros públicos (directos - convenios), privados y fiscomisionales donde se imparta atenciones de cuidado diario, actividades de juego y aprendizaje, acciones de salud y nutrición para niñas y niños entre los 45 días de nacido hasta 3 años de edad, con articulación intersectorial, siendo inclusivos e interculturales”* | *“Promote comprehensive protection in the provision of comprehensive early childhood development services, which includes public (direct - agreements), private and fiscal dependent centres where daily care, play and learning activities, health and nutrition actions are provided for girls and boys between 45 days of age and 3 years of age, with intersectoral articulation, being inclusive and intercultural”* | Technical Standard of the CDI Child Development Centre Service, Ministerial Agreement 38, Official Registry Supplement 383 (28 August 2023) |
|  | Cash transfers | Recommendations for cash transfers or subsidies to families with children in situations of poverty and vulnerability to improve child well-being | *“Créase el Bono 1000 Días, consistente en una transferencia monetaria condicionada de sesenta dólares de los Estados Unidos de América (USD 60,00), sujetos al cumplimiento de condicionalidades de corresponsabilidad”* | *“The 1000 Days Benefit is hereby created, consisting of a conditional monetary transfer of sixty United States dollars (USD 60.00), subject to compliance with co-responsibility conditions”* | 1000-day Benefit, Executive Decree 435, Official Registry Supplement 84 (15 June 2022) |
|  | Social support for vulnerable families | Recommendations to ensure the safety, well-being and equal opportunities for families in vulnerable situations, promoting their access to essential services and their full participation in society | *“Con base en la corresponsabilidad y a través de un acompañamiento diferenciado, la unidad de atención debe analizar la situación de cada familia, brinda información pertinente sobre cómo acceder a los servicios y programas sociales del Estado, realiza el seguimiento y la evaluación del progreso en la consecución de las condiciones básicas de desarrollo familiar”* | *“Based on co-responsibility and through differentiated support, the care unit must analyse the situation of each family, provide relevant information on how to access the State's social services and programmes, and monitor and evaluate progress in achieving the basic conditions for family development.”* | Technical Standard for Family Support Modality for Beneficiaries of the Human Development Benefit with Variable Component (BDHV), Ministerial Agreement 007, Official Registry 250 (14 February 2023) |
|  | Child nutrition in social programmes | Recommendations to promote child health and well-being, including nutrition in social programmes. | *“Tendrá por objeto la entrega periódica de raciones alimenticias diversificadas, en la población objetivo determinada en la Ley de Seguridad Alimentaria y Nutricional según la focalización realizada por el SELBEN”* | *“Its objective will be the periodic delivery of diversified food rations to the target population determined in the Food and Nutrition Security Law, according to the targeting carried out by SELBEN”* | “Alimentate Ecuador “Programme, Ministerial Agreement 359 (14 May 2007) |
|  | Protection from violence, abuse and neglect | Recommendations for preventing and responding to violence, abuse and neglect against children, whether at home or in the community | “*En caso de conocer de situaciones de vulneración de derechos de las adolescentes gestantes, niñas y niños menores de 3 años, deberá remitir un informe a la Directora/or Distrital quien realizará las denuncias respectivas en los organismos competentes…”.* | “*In the event of becoming aware of situations of violation of the rights of pregnant adolescents, girls and boys under 3 years of age, a report must be sent to the District Director who will make the respective complaints to the competent bodies… “*. | Family Care Service Growing with our Children, Ministerial Agreement 36, Official Registry Supplement 382 (25 August 2023) |
| **Child care** | Access to childcare | Recommendations to ensure that children have access to adequate, safe and quality childcare services without discrimination. | *“El Empleador tanto del sector público como del privado recibirá las solicitudes de las personas trabajadoras para acceder a este beneficio, pudiendo optar la dotación de dichos servicios a través de: a) Centros de cuidado diario infantil financiados con recursos públicos; b) Centros de cuidado diario infantil, creados o que se creen y manejados directamente por las instituciones del sector público o sector privado; o, c) Centros de cuidado infantil privado”.* | *“The Employer, both in the public and private sectors, will receive requests from workers to access this benefit, and may choose to provide said services through: a) Childcare centres financed with public resources; b) Childcare centres, created or to be created and managed directly by institutions in the public or private sector; or c) Private childcare centres.”* | Organic Law on the Right to Human Care, Law 0 Official Registry Supplement 309 (12 May 2023) |
|  | Parental leave policies | Recommendations to ensure the rights and conditions under which parents or legal caregivers may take time off work to care for their children in early childhood | *“La licencia de maternidad remunerada se entenderá aquel periodo de tiempo desde el nacimiento hasta el tiempo máximo que establezcan las leyes vigentes que reglan las relaciones del talento humano según corresponda”* | *“Paid maternity leave shall be understood as the period from birth to the maximum time established by current laws that regulate human talent relations as appropriate.”* | Organic Law on the Right to Human Care, Law 0 Official Registry Supplement 309 (12 May 2023) |
|  | Support for working parents | Recommendations for supporting working mothers/fathers in reconciling work life and parental responsibilities. | *“Las instituciones del Estado en las que laboren más de veinte servidoras o servidores que tengan beneficiarios deberán otorgar a estos últimos, el beneficio de guardería…”* | *“State institutions in which more than twenty male or female employees work and who have beneficiaries must grant the latter the benefit of daycare.”,* | Childcare Benefit for Children of Public Servants, Ministerial Agreement 85 Supplement to Official Registry 344 (03 July 2023) |
| **Housing** | Access to housing | Recommendations to ensure that vulnerable families and/or those with children can access decent, adequate and affordable housing within social housing programmes | *“El ente rector de hábitat y vivienda utilizará como criterios de priorización de los postulantes para subsidios e incentivos de vivienda, las siguientes vulnerabilidades:..…2. Familias monoparentales (padres o madres solos) que tengan bajo su protección y cuidado a niñas, niños y adolescentes. 3. Mujeres embarazadas….., 4. Núcleos familiares que tengan una o más personas con discapacidad….5. Núcleos familiares que tengan bajo su protección y cuidado a una o más personas con enfermedades catastróficas, enfermedades raras, enfermedades huérfanas o enfermedades de alta complejidad…..; 6. Núcleos familiares expuestos a situaciones de violencia sexual o doméstica, que consten en el Registro Único de Violencia (RUV), generado por el Ministerio de la Mujer y Derechos Humanos, o quien haga sus veces. 7. Núcleos familiares conformados por personas de pueblos y nacionalidades del Ecuador.* | *“The governing body for habitat and housing will use the following vulnerabilities as prioritisation criteria for applicants for housing subsidies and incentives: …2. Single-parent families (single fathers or mothers) that have girls, boys and adolescents under their protection and care. 3. Pregnant women…………. 4. Family units that have one or more people with disabilities………… 5. Family units that have under their protection and care one or more people with catastrophic illnesses, rare diseases, orphan diseases or highly complex diseases………. 6. Family nuclei exposed to situations of sexual or domestic violence, which are recorded in the Single Registry of Violence (RUV), generated by the Ministry of Women and Human Rights, or whoever takes its place. 7. Family nuclei made up of people from ethnic minorities and nationalities of Ecuador.* | Regulation Governing Access to Housing Subsidies and Incentives, Ministerial Agreement 12, Official Register 604 (19 July 2024) |
|  | Access to safe water and sanitation | Recommendations to ensure that all households have access to safe drinking water and adequate sanitation services. | *“El ente rector de Planificación Nacional brindará asistencia y asesoría técnica a los Gobiernos Autónomos Descentralizados en el proceso de formulación de los Planes de Ordenamiento Territorial, haciendo énfasis en la necesidad de priorización e implementación de programas o proyectos de salud, agua y saneamiento que incidan en la disminución de la desnutrición crónica infantil”* | *“The National Planning governing body will provide assistance and technical advice to the Decentralised Autonomous Governments in the process of formulating Territorial Planning Plans, emphasising the need for prioritisation and implementation of health, water and sanitation programmes or projects that have an impact on the reduction of chronic childhood malnutrition.”* | National Strategy Ecuador Grows without Malnutrition, Executive Decree 1211 Supplement 356 of the Official Register (23 December 2020) |
| **Vulnerable groups** | Children living in poverty | Recommendations for access to essential early childhood services for children living in poverty. | *“Está dirigida a mujeres gestantes y familias con niñas y niños de cero a tres años de edad, cuyo núcleo familiar se encuentra en zonas con alta prevalencia de determinantes multicausales de pobreza, pobreza extrema y vulnerabilidad.”* | *“It is aimed at pregnant women and families with children from zero to three years of age, whose family nucleus is located in areas with a high prevalence of multi-causal determinants of poverty, extreme poverty and vulnerability.”* | Family Care Service Growing with our Children, Ministerial Agreement 36, Official Registry Supplement 382 (25 August 2023) |
|  | Ethnic minorities | Recommendations for interventions in children with different ethnic identities in the access, quality and cultural adequacy of early childhood services | *“Además, para la operación de los servicios se debe considerar las características poblacionales como etnia, lengua materna, edad de la madre, padre o cuidadores, escolaridad, movilidad; y otras características como los horarios de trabajo, disponibilidad y accesibilidad a centros y espacios comunitarios; contexto comunitario y del entorno.”* | *“In addition, the operation of services must take into account population characteristics such as ethnicity, mother tongue, age of mother, father or caregivers, education, mobility, and other characteristics such as work schedules, availability and accessibility to community centres and spaces; community and environmental context.”* | Comprehensive Child Development Operational Manual “Mision Ternura”, Ministerial Agreement 3 Official Registry Special Edition 355 (17 February 2020) |
|  | Disabilities | Recommendations for inclusion, access and adaptability of early childhood programmes for children with disabilities. | *“Para la atención de niñas y niños de 1 a 3 años de edad con discapacidad leve, moderada con autonomía física y/o alertas en el proceso de desarrollo y aprendizaje, se aplicará el Protocolo para la atención integral de las niñas y niños de 0 a 3 años de edad con Discapacidad y/o alertas en el proceso de desarrollo aprendizaje”* | *“For the care of girls and boys from 1 to 3 years of age with mild or moderate disabilities with physical autonomy and/or alerts in the development and learning process, the Protocol for the comprehensive care of girls and boys from 0 to 3 years of age with disabilities and/or alerts in the development and learning process will be applied”* | Technical Standard of the CDI Child Development Centre Service, Ministerial Agreement 38, Official Registry Supplement 383 (28 August 2023) |
|  | Migrants and Refugees | Recommendations for early childhood care in contexts of forced mobility or migration. | *“Todo recién nacido(a) hijo(a) de madre que se encuentra en condición de refugiada, solicitante de asilo, retornada, migrante, desplazada interna y/o apátrida, deberá ser atendido (a) con calidad y calidez en todos los establecimientos de salud de la Red Pública Integral Salud. La atención en salud es totalmente gratuita.”* | *“Every newborn child of a mother who is a refugee, asylum seeker, returnee, migrant, internally displaced person and/or stateless person must receive quality and warm care in all health facilities of the Comprehensive Public Health Network. Health care is completely free.”* | Healthcare of Pregnant Women and Newborns in Mobility Conditions, Ministerial Agreement 98, Official Registry Supplement 589 (28 June 2024) |
|  | Children in orphanhood or single-parent homes | Recommendations for access to support services for children in single-parent families or orphans | *“Serán beneficiarios de este Bono las niñas, niños o adolescentes, comprendidos entre los 0 a los 18 años de edad que se encuentren en situación de orfandad a causa del cometimiento de los siguientes delitos en contra de su madre o progenitora: Asesinato, Femicidio, Homicidio, Violación con resultado de muerte.”* | *“The beneficiaries of this Benefit will be girls, boys or adolescents, between 0 and 18 years of age, who are orphaned due to the commission of the following crimes against their mother or parent: Murder, Femicide, Homicide, Rape resulting in death.”* | Creation of a Benefit for Children in the Event of the Violent Death of their Mother. Executive Decree 370, Official Registry Supplement 27 (23 March 2022) |
|  | Children living in rural or remote areas | Recommendations for access to essential early childhood services for children living in rural or remote areas. | *“Círculos de cuidado, recreación y aprendizaje (CCRA), atiende a familias con niñas, niños de 0 a tres años y mujeres gestantes en condición de pobreza, extrema pobreza y alta vulnerabilidad en zonas rurales con alta dispersión”* | *“Care, Recreation and Learning Circles (CCRA) serves families with children from 0 to three years old and pregnant women in conditions of poverty, extreme poverty and high vulnerability in rural areas with high dispersion”* | Comprehensive Child Development Operational Manual “Mision Ternura”, Ministerial Agreement 3 Official Registry Special Edition 355 (17 February 2020) |
|  | Children with catastrophic or rare diseases | Recommendations for access to essential early childhood services for children with serious or rare health conditions. | *“….Serán beneficiarios del bono “Joaquín Gallegos Lara” todos los menores de catorce años viviendo con VIH - SIDA.”* | *“[….] all minors under fourteen years of age living with HIV - AIDS will be beneficiaries of the Joaquín Gallegos Lara Benefit.”* | Creates Joaquín Gallegos Lara Benefit in favour of people with disabilities, Executive Decree 422 Supplement Official Registry 252 (06 August 2010) |
|  | Children victims of violence, abuse and neglect | Recommendations for access to essential services in early childhood for children who are victims of violence | *“Evaluar maltrato infantil (físico y/o psicológico) y abuso sexual en niñas y niños menores de 5 años. En toda niña o niño que se evalúe por cualquier causa, se debe investigar si existe maltrato.”* | *“Evaluate child abuse (physical and/or psychological) and sexual abuse in girls and boys under 5 years of age. In any girl or boy who is evaluated for any reason, an investigation must be carried out to determine if there is abuse.”* | Procedures for the Care of Prevalent Childhood Diseases, Ministerial Agreement 225, Official Registry Special Edition 486 (16 July 2018) |
|  | Children of mothers in prison | Recommendations for access to essential early childhood services for children whose mother is incarcerated. | *“Los CDI Públicos (directos - convenios) deben atender a niñas y niños de 1 a 3 años de edad, que convivan con sus madres privadas de libertad en los CPL, referidos por el Sistema Nacional de Atención Integral a Personas Adultas Privadas de Libertad y Adolescentes infractores (SNAI)”* | *“Public CDIs (direct-agreements) must care for girls and boys from 1 to 3 years of age, who live with their mothers deprived of liberty in the CPL, referred by the National System of Comprehensive Care for Adult Persons Deprived of Liberty and Adolescent Offenders (SNAI)”* | Technical Standard of the CDI Child Development Centre Service, Ministerial Agreement 38, Official Registry Supplement 383 (28 August 2023) |
| **Universal Policies** | Access to education | Recommendations that promote access to early childhood education. | *“Según lo establecido en la Política Pública de la Primera Infancia, el vínculo de las niños y niños en los servicios de desarrollo infantil, culmina una vez que cumplen los 36 meses de edad, luego de lo cual, se promueve su continuidad hacia los programas del Ministerio de Educación, de acuerdo a lo dispuesto en las Normas Técnicas CDI y CNH”* | *“According to the provisions of the Early Childhood Public Policy, the link between children and child development services ends once they reach 36 months of age, after which their continuity is promoted towards the Ministry of Education programmes, in accordance with the provisions of the CDI and CNH Technical Standards.”* | Comprehensive Child Development Operational Manual “Mision Ternura”, Ministerial Agreement 3 Official Registry Special Edition 355 (17 February 2020) |
|  | Access to health services | Recommendations aimed at ensuring that all children have coverage and access to health services. | *“Toda mujer tiene derecho a la atención de salud gratuita y de calidad durante su embarazo, parto y postparto, así como al acceso a programas de salud sexual y reproductiva. De igual manera se otorgará sin costo la atención de salud a los recién nacidos-nacidas y niños-niñas menores de cinco años, como una acción de salud pública, responsabilidad del Estado”.* | *“Every woman has the right to free, quality health care during pregnancy, childbirth and postpartum, as well as access to sexual and reproductive health programmes. Likewise, health care will be provided free of charge to newborns and children under five years of age, as a public health action, the responsibility of the State.”* | Law on Free Maternity and Child Healthcare, Codification 6 Supplement to Official Register 349 (05 September 2006) |
|  | Access to social protection | Recommendations aimed at ensuring that all children have coverage and access to child protection services without discrimination. | *“Asegurar que, las mujeres embarazadas, y los niños menores de 5 años, en condición de vulnerabilidad, captados para recibir atención integral, accedan a servicios de cuidado en CDI-CNH y reciban el BDH variable.”* | *“Ensure that pregnant women and children under 5 years of age, in vulnerable conditions, recruited to receive comprehensive care, have access to care services at CDI-CNH and receive the variable BDH.”* | Intersectoral Food and Nutrition Plan, Ministerial Agreement 237, Official Register Special Edition 498 (25 July 2018) |
|  | Access to childcare | Recommendations to ensure that children have access to adequate, safe and quality childcare services. | *“El Empleador tanto del sector público como del privado recibirá las solicitudes de las personas trabajadoras para acceder a este beneficio, pudiendo optar la dotación de dichos servicios a través de: a) Centros de cuidado diario infantil financiados con recursos públicos; b) Centros de cuidado diario infantil, creados o que se creen y manejados directamente por las instituciones del sector público o sector privado; o, c) Centros de cuidado infantil privado”.* | *“The Employer, both in the public and private sectors, will receive requests from workers to access this benefit, and may choose to provide said services through a) Childcare centres financed with public resources; b) Childcare centres, created or to be created and managed directly by institutions in the public or private sector; or c) Private childcare centres.”* | Organic Law on the Right to Human Care, Law 0 Official Registry Supplement 309 (12 May 2023) |
|  | Access to housing | Recommendations to ensure that vulnerable families and/or those with children can access decent, adequate and affordable housing within social housing programmes | *“El ente rector de Planificación Nacional brindará asistencia y asesoría técnica a los Gobiernos Autónomos Descentralizados en el proceso de formulación de los Planes de Ordenamiento Territorial, haciendo énfasis en la necesidad de priorización e implementación de programas o proyectos de salud, agua y saneamiento que incidan en la disminución de la desnutrición crónica infantil”* | *“The governing body of National Planning will provide assistance and technical advice to the local autonomous governments in the process of formulating territorial planning plans, emphasising the need for prioritisation and implementation of health, water and sanitation programmes or projects that have an impact on the reduction of chronic child malnutrition.”* | National Strategy Ecuador Grows without Malnutrition, Executive Decree 1211 Supplement 356 of the Official Register (23 December 2020) |
| **Intercultural approach** | Intercultural approach | Incorporating cultural elements into early childhood policies, recognising and respecting cultural diversity and adapting services to the needs of different communities and ethnic groups. | *“El diálogo de saberes entre parteras o parteros y profesionales de la salud, es un componente fundamental para garantizar su coexistencia ya que permite la articulación respetuosa de los saberes y prácticas de la partería ancestral y los conocimientos específicos de la medicina convencional.”* | *“The dialogue of knowledge between midwives and health professionals is a fundamental component to guarantee their coexistence since it allows the respectful articulation of the knowledge and practices of ancestral midwifery and the specific knowledge of conventional medicine.”* | Manual on the Articulation of Practices and Knowledge of Ancestral Midwives, Ministerial Agreement 161, Official Register 347 (06 July 2023) |
