# Supplementary material for: Addressing social determinants of health and equity in early childhood: a qualitative document analysis of national policies in Ecuador
Source: Int J Equity Health. 2026 May 29;25:180. doi: 10.1186/s12939-026-02891-2 (PMC13422072; doi:10.1186/s12939-026-02891-2)
Supplement: Supplementary file 5 — Supplementary Material 5 [file 12939_2026_2891_MOESM5_ESM.docx]

**Supplementary Table 2.** List of policy documents analysed and links to access

| Lead Institution | Year | Title of the document | Document reference | Policy document Link |
| --- | --- | --- | --- | --- |
| Ministry of Public Health | 2006 | Law on Free Maternity and Child Healthcare | Codification 6 Supplement to Official Register 349 (05 September 2006) | <http://esacc.corteconstitucional.gob.ec/storage/api/v1/10_DWL_FL/eyJjYXJwZXRhIjoicm8iLCJ1dWlkIjoiMjZjNTI4YzMtMjdlNi00OWJhLTkzZGItYzU0MmRhZDU2ZTg0LnBkZiJ9> |
|  | 2018 | Procedures for the Healthcare of Prevalent Childhood Diseases | Ministerial Agreement 225, Official Registry Special Edition 486 (16 July 2018) | <http://esacc.corteconstitucional.gob.ec/storage/api/v1/10_DWL_FL/eyJjYXJwZXRhIjoicm8iLCJ1dWlkIjoiYzg1OGNkNjQtMDRhNy00NDY5LTg2ZGQtYTI5MDI0NTdlYjUzLnBkZiJ9> |
|  | 2011 | Zero Malnutrition Project | Ministerial Agreement 175, Official Register 411 (23 March 2011) | <http://esacc.corteconstitucional.gob.ec/storage/api/v1/10_DWL_FL/eyJjYXJwZXRhIjoicm8iLCJ1dWlkIjoiNjIxZDk5ZTgtZjYzYS00ZWZjLWE3ZDItMWQzMGNmMzBjYWM5LnBkZiJ9> |
|  | 2018 | Comprehensive Child Healthcare Manual | Ministerial Agreement 238, Official Register Special Edition 499 (25 July 2018) | <http://esacc.corteconstitucional.gob.ec/storage/api/v1/10_DWL_FL/eyJjYXJwZXRhIjoicm8iLCJ1dWlkIjoiOTQwOWI1YmMtZTNmZi00YTZlLTkyNDUtNzZmYTcwNjE4MmNmLnBkZiJ9> |
|  | 2013 | Standard for Essential Obstetric and Neonatal Healthcare | Ministerial Agreement 3599 Supplement to the Official Register 39 (18 July 2013) | <http://esacc.corteconstitucional.gob.ec/storage/api/v1/10_DWL_FL/eyJjYXJwZXRhIjoicm8iLCJ1dWlkIjoiZDVkMDQ4ZDgtNTM0OS00NTE0LWI5YjAtMTgwMGUxYzZlYTdlLnBkZiJ9> |
|  | 2018 | Intersectoral Plan for Food and Nutrition | Ministerial Agreement 237, Official Register Special Edition 498 (25 July 2018) | <http://esacc.corteconstitucional.gob.ec/storage/api/v1/10_DWL_FL/eyJjYXJwZXRhIjoicm8iLCJ1dWlkIjoiZjdmZWM4MDEtMDhjYy00MmRhLTlmMTItYmEyZDM5ZjExMTZhLnBkZiJ9> |
|  | 2023 | Manual for Monitoring Nutritional Status of Iodine | Ministerial Agreement 120, Official Register 310 (15 May 2023) | <http://esacc.corteconstitucional.gob.ec/storage/api/v1/10_DWL_FL/eyJjYXJwZXRhIjoicm8iLCJ1dWlkIjoiNDYyZjhiZGMtZTVmZC00MzVmLWEzYmYtM2Y3MzUyNzc0MjYyLnBkZiJ9> |
|  | 2019 | Vaccine Manual for Immunopreventable Diseases | Ministerial Agreement 63, Official Registry Special Edition 143 (28 November 2019) | <http://esacc.corteconstitucional.gob.ec/storage/api/v1/10_DWL_FL/eyJjYXJwZXRhIjoicm8iLCJ1dWlkIjoiYzBmMDJmMDktNDM1MS00ZGU5LWE2OGUtZTBlMTk2ZWZlOGU1LnBkZiJ9> |
|  | 2023 | Manual on the Articulation of Practices and Knowledge of Ancestral Midwives | Ministerial Agreement 161, Official Register 347 (06 July 2023) | <http://esacc.corteconstitucional.gob.ec/storage/api/v1/10_DWL_FL/eyJjYXJwZXRhIjoicm8iLCJ1dWlkIjoiYzQ0YzBkYzktYjY3OS00ZjgzLWE2MTItMzJkMjRmMjMyZjk2LnBkZiJ9> |
|  | 2014 | Neonatal Metabolic Screening Test in Health Units | Ministerial Agreement 4779, Official Register 222 (09 April 2014) | <http://esacc.corteconstitucional.gob.ec/storage/api/v1/10_DWL_FL/eyJjYXJwZXRhIjoicm8iLCJ1dWlkIjoiNTA2ZDY1ZmYtMzgwMC00MjNiLWI1M2UtMTdiMWJjMmNhM2ZkLnBkZiJ9> |
|  | 2019 | Strategy for the Elimination of Mother-to-Child Transmission of HIV, Syphilis, Hepatitis B, and Chagas | Ministerial Agreement 373, Official Registry Special Edition 1004 (09 July 2019) | <http://esacc.corteconstitucional.gob.ec/storage/api/v1/10_DWL_FL/eyJjYXJwZXRhIjoicm8iLCJ1dWlkIjoiNGYzYTY3ZjEtMDQ0OC00M2YwLWEwYzAtZWEwY2I5NGM4MjY5LnBkZiJ9> |
|  | 2024 | Healthcare for Pregnant Women and Neonates in Mobility Conditions | Ministerial Agreement 98, Official Registry Supplement 589 (28 June 2024) | <http://esacc.corteconstitucional.gob.ec/storage/api/v1/10_DWL_FL/eyJjYXJwZXRhIjoicm8iLCJ1dWlkIjoiMDc2MzI2MDAtMThiZS00MWQxLTg5YmMtOTY5NjNjMDRiNjNmLnBkZiJ9> |
|  | 2009 | National Breastfeeding Policy | Ministerial Agreement 675, Official Registry 47 (15 October 2009) | <http://esacc.corteconstitucional.gob.ec/storage/api/v1/10_DWL_FL/eyJjYXJwZXRhIjoicm8iLCJ1dWlkIjoiZTliOWQ2Y2UtNzliNi00NzVkLTk1YzktY2UwMWM2OTU3M2JlLnBkZiJ9> |
|  | 2008 | National Plan for Reducing Maternal and Neonatal Mortality | Ministerial Agreement 474 (20 August 2008) | <https://www.salud.gob.ec/catalogo-de-normas-politicas-reglamentos-protocolos-manuales-planes-guias-y-otros-del-msp/> |
| Ministry of Economic and Social Inclusion | 2023 | Family Care Service Growing with our Children | Ministerial Agreement 36, Official Registry Supplement 382 (25 August 2023) | <http://esacc.corteconstitucional.gob.ec/storage/api/v1/10_DWL_FL/eyJjYXJwZXRhIjoicm8iLCJ1dWlkIjoiYzFlNDkwZGQtMDJjMS00YTNkLWFjNGUtMDAyMWM5NzAxZjczLnBkZiJ9> |
|  | 2023 | Technical standard for the Child Development Centre Service | Ministerial Agreement 38 Official Registry Supplement 383 (28 August 2023) | <http://esacc.corteconstitucional.gob.ec/storage/api/v1/10_DWL_FL/eyJjYXJwZXRhIjoicm8iLCJ1dWlkIjoiNGE3ZTliMmUtZjVjNy00M2YzLTgyYTctZjk3Y2Q0OWU3MzRhLnBkZiJ9> |
|  | 2019 | National Plan for the Prevention of violence against children and adolescents | Ministerial Agreement 40, Official Register 422 (06 February 2019) | <http://esacc.corteconstitucional.gob.ec/storage/api/v1/10_DWL_FL/eyJjYXJwZXRhIjoicm8iLCJ1dWlkIjoiMzNkZGIwNTYtOTU1MC00MmFmLWFiY2YtMTEzMDk0M2RlYmI1LnBkZiJ9> |
|  | 2018 | Human development Benefit aimed at Beneficiaries with Minor Children | Ministerial Agreement 007, Official Registry 250 (14 February 2023) | <http://esacc.corteconstitucional.gob.ec/storage/api/v1/10_DWL_FL/eyJjYXJwZXRhIjoicm8iLCJ1dWlkIjoiYzJmNmE2ZGQtOTJlNi00ZDViLWI3M2QtYjAwYTNhNjlmNmVlLnBkZiJ9> |
|  | 2022 | Benefit for Children in case of Violent Death of their Mother | Executive Decree 370, Official Registry Supplement 27 (23 March 2022) | <http://esacc.corteconstitucional.gob.ec/storage/api/v1/10_DWL_FL/eyJjYXJwZXRhIjoicm8iLCJ1dWlkIjoiNzU5ZjhiOTEtMTY2YS00NmM2LWE0MDQtM2U2YWFiODM5OWM4LnBkZiJ9> |
|  | 2023 | Family Support Modality for Beneficiaries of the Human Development Benefit with Variable Component (BDHV) | Ministerial Agreement 007, Official Registry 250 (14 February 2023) | <http://esacc.corteconstitucional.gob.ec/storage/api/v1/10_DWL_FL/eyJjYXJwZXRhIjoicm8iLCJ1dWlkIjoiYzJmNmE2ZGQtOTJlNi00ZDViLWI3M2QtYjAwYTNhNjlmNmVlLnBkZiJ9> |
|  | 2010 | Joaquín Gallegos Lara Benefit for People with Disabilities | Executive Decree 422 Supplement Official Registry 252 (06 August 2010) | <http://esacc.corteconstitucional.gob.ec/storage/api/v1/10_DWL_FL/eyJjYXJwZXRhIjoicm8iLCJ1dWlkIjoiZjg5ZjQ5MDgtNGM3My00MGU3LWIyZDktN2Q0MmM1NGIwNzIwLnBkZiJ9> |
|  | 2007 | "Aliméntate Ecuador" program | Ministerial Agreement 359 (14 May 2007) | <https://www.salud.gob.ec/catalogo-de-normas-politicas-reglamentos-protocolos-manuales-planes-guias-y-otros-del-msp/> |
| Ministry of Labour | 2024 | Breastfeeding Support Rooms in the Workplace | Ministerial Agreement 2, Official Register 642 (12 September 2024) | <http://esacc.corteconstitucional.gob.ec/storage/api/v1/10_DWL_FL/eyJjYXJwZXRhIjoicm8iLCJ1dWlkIjoiNjliYzJhODMtZDZjOC00MzBhLTk4MDktZmQyOTIzODg3OTFmLnBkZiJ9> |
|  | 2023 | Childcare for Children of Public Servants | Ministerial Agreement 85 third Supplement to Official Registry 344 (03 July 2023) | <http://esacc.corteconstitucional.gob.ec/storage/api/v1/10_DWL_FL/eyJjYXJwZXRhIjoicm8iLCJ1dWlkIjoiMWE2MWQ2M2EtZWM5OC00N2NhLWI0Y2QtODIwMTU4ZjBlNzExLnBkZiJ9> |
|  | 2023 | Organic Law on the Right to Human Care | Law 0 Official Registry Supplement 309 (12 May 2023) | <http://esacc.corteconstitucional.gob.ec/storage/api/v1/10_DWL_FL/eyJjYXJwZXRhIjoicm8iLCJ1dWlkIjoiMGJmMWM2MmMtNTViMy00NDA0LTk2ZWYtMjBmNDRmODA1MDU4LnBkZiJ9> |
| Ministry of Housing | 2024 | Regulation for Access to Housing Subsidies and Incentives | Ministerial Agreement 12, Official Register 604 (19 July 2024) | <http://esacc.corteconstitucional.gob.ec/storage/api/v1/10_DWL_FL/eyJjYXJwZXRhIjoicm8iLCJ1dWlkIjoiMjNhNjEyMjYtMTkyZS00YTY5LTg3OWMtMmJmNTlhYmMyNzM3LnBkZiJ9> |
| Technical Secretariat | 2020 | Comprehensive Child Development Operational Manual “Misión Ternura” | Ministerial Agreement 3 Official Registry Special Edition 355 (17 February 2020) | <https://esilecstorage.s3.amazonaws.com/biblioteca_silec/REGOFORIGINAL/2020/DC20B0AF844C29371681358CCE23A9BBF800324A.pdf> |
|  | 2020 | National Strategy Ecuador Grows without Malnutrition | Executive Decree 1211 Supplement to the Official Register 356 (23 December 2020) | <https://esilecstorage.s3.amazonaws.com/biblioteca_silec/REGOFORIGINAL/2020/FE777BA453C30929464EDF6AA83735E2DCAB2A26.pdf> |
|  | 2022 | 1000 Days Benefit | Executive Decree 435 Official Registry Third Supplement 84 (15 June 2022) | <http://esacc.corteconstitucional.gob.ec/storage/api/v1/10_DWL_FL/eyJjYXJwZXRhIjoicm8iLCJ1dWlkIjoiMDBhNDM5NjYtYjEzMC00MmQyLWI2YmYtNmI1YjIxZjlkMThmLnBkZiJ9> |
